# Evidence from plutonic xenoliths for magma differentiation, mixing and storage in a volatile-rich crystal mush beneath St. Eustatius, Lesser Antilles

**DOI:** 10.1007/s00410-019-1576-4

**Published:** 2019-05-06

**Authors:** George F. Cooper, Jon D. Blundy, Colin G. Macpherson, Madeleine C. S. Humphreys, Jon P. Davidson

**Affiliations:** 1 Department of Earth Sciences, Durham University, Science Labs, Durham, DH1 3LE UK; 2 School of Earth Sciences, University of Bristol, Wills Memorial Building, Bristol, BS8 1RJ UK

**Keywords:** Lesser Antilles, St. Eustatius, Cumulate, Xenolith, Melt inclusions, Crystal mush

## Abstract

Quantifying the storage conditions and evolution of different magmatic components within sub-volcanic plumbing systems is key to our understanding of igneous processes and products. Whereas erupted magmas represent a portion of the eruptible volcanic system, plutonic xenoliths provide a complementary record of the mushy roots of the plumbing system that cannot be mobilised easily to form lavas and consequently offer a unique record of magma diversity within the sub-volcanic plumbing system. Here, we present a detailed petrological and geochemical study of erupted plutonic xenoliths from the island of Sint Eustatius (Statia), in the northern Lesser Antilles volcanic arc. The plutonic xenoliths are predominantly gabbroic, but vary in texture, mineral assemblage and crystallisation sequence. We report major, trace and volatile (H_2_O and CO_2_) concentrations of xenolith-hosted melt inclusions (MIs) and interstitial glass. The MIs have a very large range in major element (49–78 wt% SiO_2_ and 0.1–6.1 wt% MgO) and trace element concentration (72–377 ppm Sr, 32–686 ppm Ba, 39–211 ppm Zr). Their chemistry varies systematically with host phase and sample type. Significantly, it shows that (1) plutonic xenoliths record a complete differentiation sequence from basalt to rhyolite (2) apatite, but not zircon, saturation was reached during crystallisation, (3) amphibole breakdown reactions play a role in the genesis of shallow gabbronorite assemblages, and (4) mixing between crystal cargos and multiple discrete bodies occurred. Residual melt volatile contents are high (≤ 9.1 wt% H_2_O and ≤ 1350 ppm CO_2_), returning volatile saturation pressures of 0–426 MPa. Multiple reaction geobarometry and experimental comparisons indicate that equilibration took place in the upper-middle crust (0–15 km). We infer that the Statia plutonic xenoliths represent portions of a large heterogeneous crystal mush within which a great diversity of melts was stored and mixed prior to eruption. Our data show that compositional variations in magmatic plumbing systems exceed those observed in volcanic products, a likely consequence of the blending that occurs prior to and during eruption.

**Electronic supplementary material:**

The online version of this article (10.1007/s00410-019-1576-4) contains supplementary material, which is available to authorized users.

## Introduction

The final, erupted products of arc volcanoes record the integrated history of magmatic differentiation within long-lived magmatic plumbing systems. As a result, there is a potential disconnect between erupted materials and individual components of the volumetrically larger plumbing system, such that information regarding the diversity of magmatic processes and geochemistry may be lost. Plutonic xenoliths, brought to the surface during eruptions, provide a means to bridge this disconnect and to investigate the range of components present in the sub-volcanic crust. Deposits from the Quill on the island of Sint Eustatius, Lesser Antilles, contain an abundance and large variety of plutonic xenoliths. These xenoliths may represent portions of crystal mush, crystallised portions of melt-rich dominant bodies, cumulate residues from crystal fractionation, or fragments of older igneous rock and thus have a strong potential to record processes from parts of the magmatic plumbing systems in which melts are generated, pass through and stored. Here, we demonstrate the additional insights that plutonic xenoliths can yield and explore whether the range of magma types and compositions present within a magmatic plumbing system mirror those erupted at the surface.

The chemistry of crystals and melts from plutonic xenoliths can be used to place constraints on the conditions and depth of crystallisation of the xenoliths. Previous studies have suggested that the majority of plutonic xenoliths from the Lesser Antilles represent samples of mid-upper crustal storage regions (Camejo-Harry et al. 2018; Cooper et al. 2016; Stamper et al. 2014; Tollan et al. 2012). Evidence from volcanic melt inclusions (Barclay et al. 1998), mineral geobarometers (Stamper et al. 2014) and experimental studies (Martel et al. 1999, 1998; Melekhova et al. 2015; Pichavant et al. 2002a, b; Pichavant and Macdonald 2007) suggest that Lesser Antilles magmas are stored within these mid-upper crustal reservoirs prior to eruption, although prior differentiation of primitive basalts probably occurs in the lower crust (Melekhova et al. 2015). Plutonic xenoliths from Grenada (Stamper et al. 2014), Bequia (Camejo-Harry et al. 2018) and Martinique (Cooper et al. 2016) provide textural and geochemical evidence for open system processes such as disequilibria, the involvement of crystal cargoes and percolating reactive melts. Therefore, there is an intimate petrogenetic relationship between erupted magmas and the plutonic xenoliths they entrain. The nature of this relationship provides insights into differentiation processes and pre- eruptive storage conditions of the final erupted volcanic products.

The volcanic products of the Lesser Antilles provide petrographic and geochemical evidence for mixing of melts and crystals prior to eruption. The volcanic products commonly contain a mixture of crystals derived from different portions of the magmatic system (phenocrysts, xenocrysts and antecrysts). Xenocrysts and antecrysts may be in the form of crystal clots and disaggregated cumulate material and provide direct evidence for the mechanical mixing of crystals and melt. These crystal populations may be difficult to distinguish if a magmatic system has a narrow range of geochemical variations and/or storage conditions, which would be reflected in a narrow range in crystal compositions. Therefore, melt inclusions (MIs) in volcanic rocks are commonly studied to capture geochemical variations and volatile contents (particularly H_2_O and CO_2_) of arc magmas (Wallace 2005) to place constraints on pre-eruptive magma storage conditions (Blundy and Cashman 2008; Liu et al. 2006) and the movement of volatiles through magmatic plumbing systems (Johnson et al. 2008; Mann et al. 2013; Roberge et al. 2009). Early MI data from volcanic rocks erupted in the Lesser Antilles suggested an overall increase in melt water contents from 1 to 2 wt% in primary/parental basalts to ~ 3 wt% in basaltic andesites and ≥ 5 wt% in silicic melts (Macdonald et al. 2000).

MIs in volcanic rocks may only represent the most recent magma storage conditions or mixing episode, and therefore, information on magmatic process occurring prior to final pre-eruptive ascent is lost. Conversely, melt inclusions hosted in plutonic xenoliths provide a novel means to capture the diversity of magmas, and magmatic processes during the whole evolution over a range of storage conditions within arc crust. However, there have been comparatively few studies of MIs contained within plutonic xenoliths and/or cumulates (Schiano et al. 2004; Webster and Rebbert 2001; Yanagida et al. 2018). Here, we present a detailed petrological, mineralogical and geochemical dataset, with a focus on the chemistry and volatile contents of melt inclusions and interstitial glass from plutonic xenoliths from a single, arc volcano. We use these data to establish a model of the sub-volcanic plumbing system beneath the island and to trace the diverse range of melts which are present therein. We show that plutonic xenolith-hosted MIs record an entire differentiation sequence from basalt to rhyolite and that the associated volcanic rocks represent mixes of crystal cargoes and variable melt compositions. Consequently, compositional variations in magmatic plumbing systems are likely to be greater than can be observed by studying the volcanic products alone.

## Geological background

**Fig. 1:**
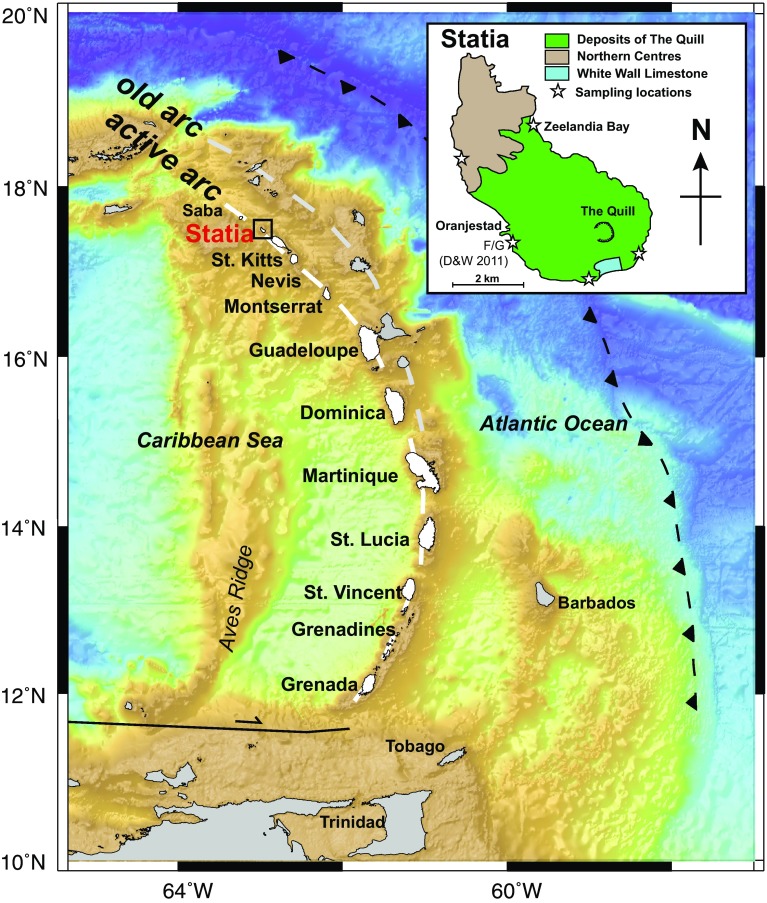
Bathymetric map of the Lesser Antilles volcanic arc. Location of Statia is shown in red. Inset sketch map of Statia shows locations of the three main stratigraphic units and sample localities used in this study

The 750 km-long Lesser Antilles Volcanic Arc is located along the eastern margin of the Caribbean Plate as the result of the relatively slow (~ 2 cm/year) westward subduction of Atlantic oceanic lithosphere (North and South American plates). In the north, the arc shows an older segment to the east and currently active arc to the west. This apparent westward jump has been attributed to flattening of the subducting slab and likely occurred ~ 7 Ma (Bouysse and Westercamp 1990). St. Eustatius (Statia) is located in the active, northern segment of the arc (Fig. 1). Westermann and Kiel (1961) and later Roobol and Smith (2004) provide detailed descriptions of the geology of the island. In summary, it comprises three major units (Fig. 1). The youngest deposits are those of the Quill, a single volcanic cone which dominates the southern end of the island (Fig. 1) and was active from 22.24 to 1.55 kyr (Roobol and Smith 2004). Deposits from the Quill are dominated by pyroclastic material, which is well exposed in cliffs on the northeast and southwest shorelines. Magmas erupted from the Quill are dominantly andesitic, although compositions ranging from basalt to rhyolite also occur. In addition to the Quill, volcanic activity was present in the Northern Centres, which are composed of five extinct volcanic centres and originally constituted an independent volcanic island, until pyroclastic deposits from the younger Quill volcano linked the two islands (Roobol and Smith 2004). The age of the Northern Centres is not well constrained but is thought to be < 1 Ma. Intermediate in age between the Quill and Northern Centres is the White Wall-Sugar Loaf Ridges at the south end of the island (Fig. 1), consisting of interstratified subaqueous volcaniclastic deposits and shallow-water limestones. This succession has been uplifted and tilted by the intrusion of a dome into the southern flank of the Quill.

Volcanic rocks erupted from the Quill display one of the broadest compositional ranges of any arc volcano, from 52.0 to 72.3 wt% SiO_2_, and can be classified as low-K and high-Ca calc-alkaline (Roobol and Smith 2004). The dominant mineralogy of almost all erupted rocks is plagioclase, orthopyroxene, clinopyroxene and Fe–Ti oxides, with sparse amphibole and olivine. The volcanic rocks contain textural evidence for amphibole breakdown, with amphibole containing reaction rims of Fe–Ti oxide and pyroxene, or completely replaced. Plagioclase glomerocrysts and inclusions of gabbroic assemblages (plagioclase + clinopyroxene + orthopyroxene + Fe–Ti oxides) are common (Roobol and Smith 2004). Previous geochemical, isotopic and petrological studies have suggested that the range of compositions found at the Quill is largely driven by fractional crystallisation, with limited crustal contamination (Davidson and Wilson, 2011; Roobol and Smith, 2004). Here, we present melt inclusion data from plutonic xenoliths and a basaltic andesite: sample SE8247A from section F/G (Fig. 1) in Davidson and Wilson (2011) and section XII–XV of Roobol and Smith (2004).

Statia, along with other volcanic islands in the Lesser Antilles, is exceptional with respect to the abundance of erupted plutonic xenoliths, which form the basis of this study (Online Resource 1). Many of the plutonic xenoliths were sampled ex situ, where they have weathered out and become concentrated beneath cliff faces, but most likely originated from the pyroclastic deposits of the Quill, where they are very commonly found on the flanks. Only one xenolith (sample EU95) was sampled on a beach within the Northern Centres and may have originated from this older volcanic activity. In contrast to the lavas, the majority of plutonic xenoliths contain amphibole, along with plagioclase, olivine, clinopyroxene, Fe–Ti oxides and rare orthopyroxene. The high modal abundance of amphibole in Lesser Antilles plutonic xenoliths was first described by Arculus and Wills (1980), and is a characteristic of plutonic xenoliths from along the arc (Melekhova et al. 2019). Geochemical trends delineated by the volcanic rocks clearly demonstrate that fractionation of the observed cumulate assemblage, as preserved in some plutonic xenoliths, exerts a strong control on the generation of magmas erupted from the Quill (Davidson and Wilson 2011; Roobol and Smith 2004).

## Analytical techniques

Whole-rock major (SiO_2_, TiO_2_, Al_2_O_3_, Fe_2_O_3_, MnO, MgO, CaO, Na_2_O, K_2_O, P_2_O_5_) and selected trace elements (V, Cr, Rb, Nb, Sr, Y, Zn, Co, Ni, Ba) were analysed by X-Ray Fluorescence spectrometry using a Siemens SRS 3000 sequential XRF spectrometer at the University of Auckland. Further whole-rock trace element analysis was carried out by solution ICP-MS on a ThermoScientific X-Series 2 ICP-MS at Durham University. W-2, BHVO-1 and AGV-1 standards were used to monitor accuracy and precision. Accuracy was typically within ± 5% of the reference value and precision < 3% 2 sd.

The major element concentrations (SiO_2_, TiO_2_, Al_2_O_3_, Fe_2_O_3_, MnO, MgO, CaO, Na_2_O, K_2_O, Cr_2_O_3_, NiO) of minerals were analysed with a Cameca SX100 electron microprobe at the University of Bristol with a 20 kV accelerating voltage, 20 nA beam current and a 1 μm spot size. The instrument was calibrated using synthetic oxide, mineral and metal standards. Typical 3σ uncertainties are < 0.1 wt% for Mg, K, Ti, Mn, Cr, Ni; < 0.2 wt% for Na and Ca; < 0.4 wt% for Si, Al, Fe.

Melt inclusions within Lesser Antilles plutonic xenoliths are rare, but were identified and analysed in all major mineral phases: olivine, plagioclase, amphibole, clinopyroxene, orthopyroxene and magnetite. Melt inclusions were located with a petrographic microscope under transmitted and reflected light prior to SIMS analysis (Online Resource 2). Glassy melt inclusions ranged in size from < 10 to 200 μm (only MIs > 20 μm were analysed) and shapes varied from spherical to more angular when hosted in plagioclase. Gas bubbles were observed in a number of the MIs typically < 10% volume fraction. Small Fe–Ti oxides were observed in a number of MIs (Online Resource 2). In the majority of samples, MIs showed no signs of post-entrapment leakage. However, a number of MIs showed evidence for post-entrapment crystallisation, particularly evident in sample EU95 (gabbronorite) in which a number of MIs contained plagioclase microlites. Melt inclusions with obvious signs of crystallisation were excluded from analyses. All presented MI data are the original analyses and have not been corrected for post-entrapment crystallisation. Other glass varieties include vesiculated pockets of interstitial glass between crystals (Online Resource 2), and glass embayments. Melts (inclusion and interstitial) from 11 plutonic xenolith samples were analysed (Online Resource 1).

Prior to microbeam analysis, relevant portions of five polished thin section samples were cut into 24 mm rounds. 3 mm diameter discs, including melt inclusions and their host crystal, were drilled out of a further six polished thin sections. The 3 mm sections were then pressed into indium contained within 24 mm diameter Al holders. All samples were gold coated prior to analysis. Melt inclusions and interstitial glass were analysed by secondary ion mass spectrometry (SIMS) at the NERC ion microprobe facility at the University of Edinburgh using a Cameca IMS-4f instrument with a 15 kV (nominal) primary beam of O^−^ ions. Beam current was ~ 5 nA, resulting in a spot size at the sample surface of ~ 15 μm diameter. For CO_2_ analyses, the instrument was configured for high mass resolving power to ensure separation of 12C^+^ and 24 Mg^2+^ peaks. A secondary accelerating voltage of 4500 V with a − 50 V offset and a 25 μm image field was used. The isotopes ^12^C, ^24^Mg/2, ^26^Mg, ^30^Si were measured. Calibration was carried out on a range of basaltic glasses (S2-3, S4-13 from Pichavant et al. (2013) and 17-2 from Pichavant et al. (2009)) from with CO_2_ contents < 0.25 wt%, and standards were monitored throughout the day. Uncertainties, based on repeat analyses of a basaltic glass standard (17-2), are 5.3% relative 2 sd precision and 0.3% relative accuracy on CO_2_, and 3.6% relative 2 sd precision and a 5.0% relative accuracy on MgO. For H_2_O and trace element analyses, a secondary accelerating voltage of 4500 V with -75 V offset and a 25 μm image field was used. The isotopes ^1^H, ^7^Li, ^11^B, ^19^F, ^26^Mg, ^35^Cl, ^30^Si, ^42^Ca, ^44^Ca, ^45^Sc, ^47^Ti, ^84^Sr, ^85^Rb, ^88^Sr, ^89^Y, ^90^Zr, ^93^Nb, ^133^Cs, ^138^Ba, ^139^La, ^140^Ce, and ^149^Sm were measured. H_2_O calibration was done using ^30^Si-normalised ratios was carried out on a range of basaltic glass standards (S2-3, S4-13 and S5-14 of Pichavant et al. (2013) with 0–4 wt% H_2_O. H_2_O uncertainties, based on repeat analyses of a basaltic glass standard (S2-3), are 1.9% 2 sd precision. Precision of trace elements was < 5% 2 sd, apart from Sc (5.8%), Sm (6.1%) and Cs (18.0%). Accuracy of all trace elements was within ± 3% of the published values, apart from F (+ 5.2%) and Cs (+ 4.9%). Following analysis by ion microprobe, major element concentrations were analysed with a Cameca SX100 electron microprobe at the University of Bristol. The gold coat was removed and samples were carbon coated. Analyses of glass (SiO_2_, TiO_2_, Al_2_O_3_, Fe_2_O_3_, MnO, MgO, CaO, Na_2_O, K_2_O, P_2_O_5_, Cr_2_O_3_, NiO, Cl) were made with a 20 kV accelerating voltage, a 4 nA beam current with a 5 μm defocused beam to minimise alkali loss (Humphreys et al. 2006). Major elements were calibrated using a range of synthetic oxide, mineral and metal standards. SiO_2_ determined by EMPA was used as an internal standard for the prior SIMS analyses. Typical 3σ uncertainties are < 0.1 wt% for Mg, K, Ti, Mn, Cr, Ni, P, Cl; < 0.4 wt% for Na, Al, Ca and Fe; < 0.7 wt% for Si.

## Results

### Petrography of plutonic xenoliths

Coarse-grained intrusive igneous rocks used in this study are collectively termed plutonic xenoliths and classified using the scheme of Streckeisen (1976). We use the term cumulate only if the bulk composition, texture and mineral chemistry are consistent with a subtractive assemblage. In contrast, some plutonic xenoliths may represent aliquots of magma that have solidified without movement of crystals relative to the host melt and we term these non-cumulate gabbros. These samples are often ‘mushy’ with some patches of interstitial glass, with or without microlites, present between crystals.

**Fig. 2:**
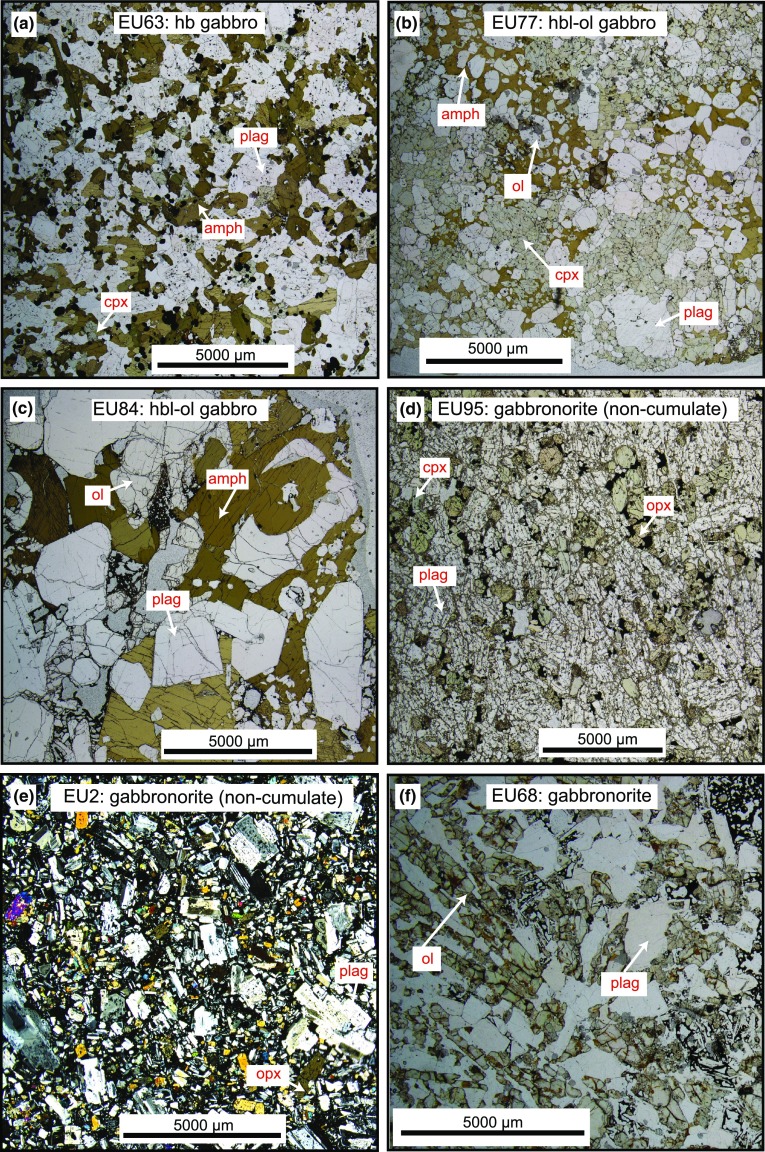
Example photomicrographs of plutonic xenolith types from which melt inclusions were analysed. **a** Hornblende gabbro (EU63) with equiangular plagioclase, clinopyroxene and amphibole. **b** Hornblende-olivine gabbro (EU77) containing amphibole oikocrysts with inclusions of olivine, plagioclase and clinopyroxene. **c** Coarse-grained hornblende-olivine gabbro cumulate (EU84) with no clinopyroxene. Interstitial melt present. **d** Amphibole-free non-cumulate gabbronorite (EU95). **e** Non-cumulate gabbronorite (EU2) with a mushy texture and intergranular melt. **f** Olivine-bearing gabbronorite (EU68) with harrisitic texture (non-cumulate)

**Fig. 3:**
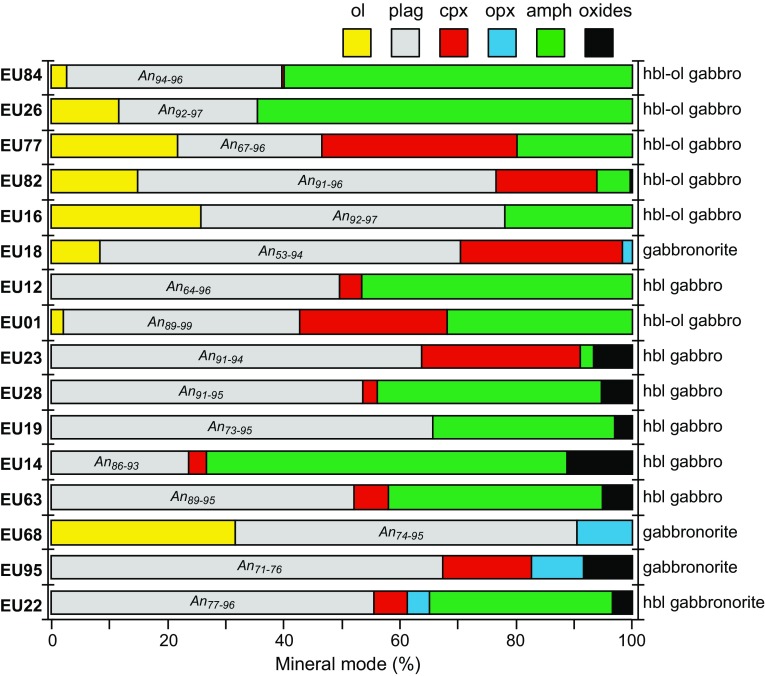
Modal proportions of mineral phases within Statia xenoliths (excluding interstitial glass). Modes determined by point counting thin sections. Samples are listed from top to bottom by decreasing Mg# [100 Mg/(Mg + Fe^2+^)] of olivine followed by Mg# of clinopyroxene Nomenclature from Streckeisen (1976)

**Fig. 4:**
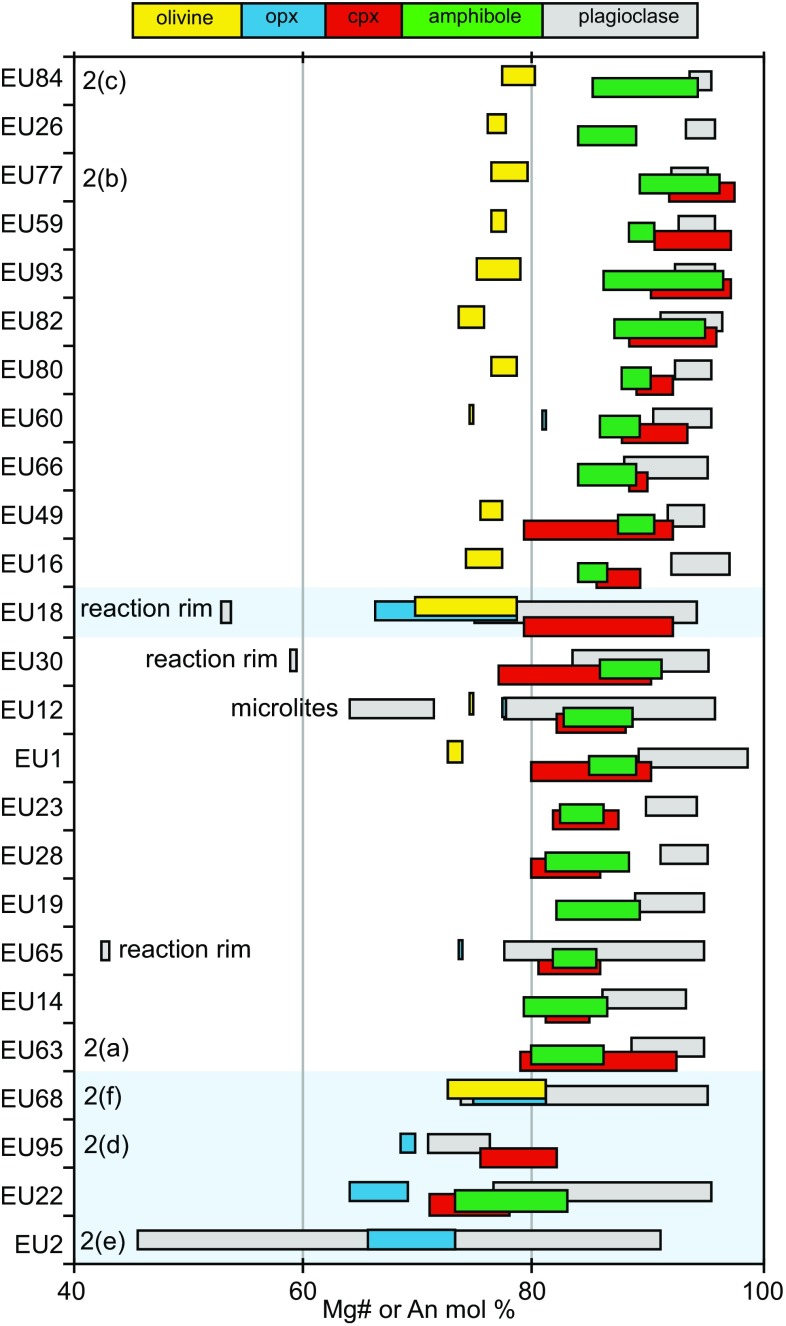
Summary of phase compositions for all analysed plutonic xenoliths: Mg# [100 Mg/(Mg + Fe^2+^)] of olivine, clinopyroxene, orthopyroxene, amphibole, and An (mol%) of plagioclase. Samples are ordered based on Mg# of olivine followed by Mg# of clinopyroxene. Samples shaded in light blue represent non-cumulate xenoliths. The six samples whose photomicrographs feature in Fig. 2 are labelled

Statia plutonic xenoliths display a large range of textures (Fig. 2) but share the same general mineral assemblages (Fig. 3) as those recorded on the other islands in the Lesser Antilles (Arculus and Wills 1980; Camejo-Harry et al. 2018; Cooper et al. 2016; Kiddle et al. 2010; Melekhova et al. 2017, 2015, 2019; Stamper et al. 2014; Tollan et al. 2012). Cumulate xenoliths are very coarse grained (≤ 1 cm) and dominated by unzoned calcic plagioclase and hornblende (Figs. 2, 3). Clinopyroxene and, to a lesser extent, olivine and Fe–Ti oxides are widespread (Fig. 3). Interstitial glass containing microlites is commonly present, may be vesiculated, and is up to 27% of the total volume. Orthopyroxene is common only in non-cumulate gabbros, which, in general, are finer grained and contain minerals that display some degree of compositional zonation (Fig. 4). The relative crystallisation sequence was determined by textural observations, i.e. a phase contained as an inclusion was assumed to have crystallised prior to its host phase. The crystallisation sequence is variable between sample types, however, when present, olivine is always the first phase to crystallise. The plutonic xenoliths can be classified as hornblende gabbros, hornblende-olivine gabbros, gabbronorites, and hornblende gabbronorites. A number of gabbronorite and hornblende gabbronorite plutonic xenoliths have a non-cumulate origin.

### Xenolith types

*Hornblende gabbros* (plag + amph ± cpx ± oxide) have grain sizes from 0.5 to 5 mm and are dominated typically by subhedral–euhedral unzoned plagioclase and amphibole crystals (e.g. Fig. 2a), with the exception of EU23, where the proportion of clinopyroxene is greater than amphibole. In general, mesocumulate and heteradcumulate textures (Wager et al. 1960) are seen and interstitial glass may be present in variable proportions. Plagioclase, alongside magnetite (found as both inclusions and interstitial grains), are the first phase to crystallise (apart from EU14, where amphibole crystallises first). In the majority of samples, amphibole appears next in the crystallisation sequence, followed by clinopyroxene. This sequence is unusual in Lesser Antilles cumulates, where amphibole appears after clinopyroxene (Cooper et al. 2016; Melekhova et al. 2017, 2015; Stamper et al. 2014; Tollan et al. 2012).

Hornblende-olivine gabbros (ol + plag + amph ± cpx ± oxide) are the most common cumulate type and display a range of heteradcumulate and mesocumulate textures (Fig. 2b, c). Grain sizes (1–10 mm) are generally greater than in hornblende gabbros and all contain some degree of interstitial glass. Unzoned, subhedral to euhedral plagioclase and amphibole dominate the mineral assemblage (Fig. 2c), with subordinate olivine and clinopyroxene (when present). In contrast to hornblende gabbros, oxides are rare. A number of samples (e.g. EU16, EU26, EU84) have clinopyroxene-free assemblages (e.g. Fig. 2c). The island of Bequia also has plutonic xenoliths with clinopyroxene-free assemblages (Camejo-Harry et al. 2018), but these are not found elsewhere in the Lesser Antilles (Cooper et al. 2016; Melekhova et al. 2017, 2015; Stamper et al. 2014; Tollan et al. 2012). Later stage amphibole forms oikocrysts, enclosing a troctolite assemblage of olivine and plagioclase chadacrysts. Sample EU26 is an exception as this also contains amphibole that crystallised before plagioclase. When present, clinopyroxene may crystallise before or after plagioclase, but always prior to amphibole. In some cases, clinopyroxene is seen breaking down and being replaced by late-stage amphibole, a process often referred to as uralitization.

Gabbronorites (plag + opx ± cpx ± ol ± oxide) are texturally diverse with variable grain size (< 1 to 5 mm) between samples, but equigranular within samples (Fig. 2d, e). Plagioclase is the dominant phase and commonly displays oscillatory zoning. Both olivine-bearing and olivine-free types occur. Where olivine is present, it is the first phase to crystallise. Sample EU68 (Fig. 2f) displays a spectacular harrisitic texture (Emeleus et al. 1996; Wadsworth, 1961; Wager et al. 1960). This is the only example of this type of cumulate xenolith in the Lesser Antilles and may represent rapid crystallisation, under conditions of olivine supersaturation or strong undercooling of the magma (O’Driscoll et al. 2006). The pyroxenes in two samples (EU2 and EU95) contain melt inclusions, which were targeted in this study. The mushy texture and oscillatory plagioclase zoning present in these two samples are suggestive of a plutonic (non-cumulate) origin (Fig. 2d, e). Accessory apatite is present in a number of gabbronorites.

Hornblende gabbronorites (plag + amph + cpx + opx ± oxide) are less common, but similar in texture to hornblende gabbros with grain sizes between 1 and 10 mm. A finer grained (< 1 mm) ‘mushy’ example (EU65) may represent a non-cumulate variety of this assemblage. Plagioclase, which dominates the assemblage, is moderately zoned from core to rim. The crystallisation sequence varies, with amphibole appearing either prior to plagioclase or late-stage. Clinopyroxene appears before plagioclase, and typically co-crystallises with orthopyroxene. Oxides appear throughout the crystallisation sequence.

### Mineral chemistry

The range in Mg number (expressed as molar % Mg/[Mg + Fe^2+^]) of olivine, clinopyroxene, orthopyroxene and amphibole, and the anorthite content (as mol  % An) of plagioclase of all studied plutonic xenoliths are summarised in Fig. 4. The Fe^2+^ was determined by stoichiometry. Below we discuss the major element variations within each mineral phase.

**Fig. 5:**
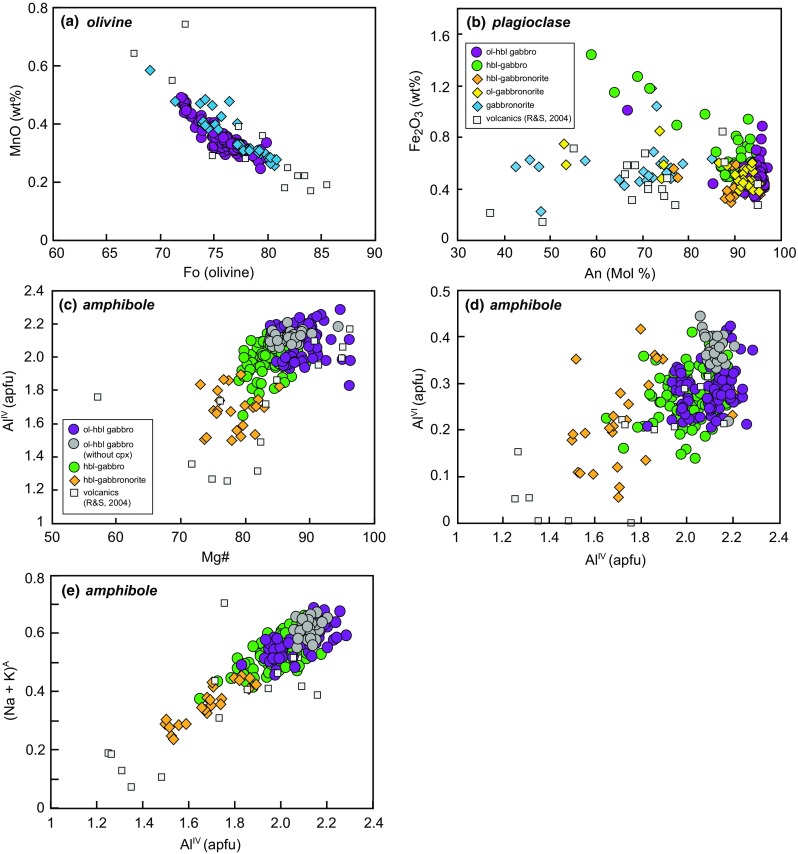
Major element mineral compositions from plutonic xenoliths and volcanic rocks. **a** Olivine Fo versus MnO (wt%) to highlight the range in compositions in plutonic xenoliths and volcanics. **b** Plagioclase An (Mol %) versus Fe_2_O_3_ (wt%). Note the divergent trends suggestive of different redox conditions. **c** Amphibole Mg# versus Al^IV^ (apfu). **d** Amphibole Al^IV^ versus Al^VI^ to show the pressure-sensitive Al-Tschermak exchange

Olivine is present in ~ 70% of studied samples and spans a relatively narrow range in composition (Mg# 69–80 = mol% forsterite, Fo) in cumulate samples (Fig. 4). Olivine from individual samples is typically homogeneous (< 4 mol% variation in Fo) apart from orthopyroxene-bearing samples EU68 and EU18 (~ 9 mol% variation in Fo). The NiO content of olivine across all samples is low (≤ 0.11 wt%), as is CaO (0.09–0.19 wt%) and these elements show no correlation with Fo content. MnO ranges from 0.25 to 0.58 wt% and is negatively correlated with Fo (Fig. 5a). Olivine from basaltic andesites, andesites and dacites from the Quill cover a larger range (Fo_65–86_) in composition (Fig. 5a). The olivines from the dacites are likely xenocrystic in origin (Roobol and Smith 2004).

Plagioclase is ubiquitous and modally dominant in all but two samples (EU26 and EU14; Fig. 3). In general, plagioclase in cumulate xenoliths is highly calcic (An_86–99_) and relatively unzoned (range of 2–9 mol% An, Fig. 3). Occasionally, lower mol% An plagioclase rims are present, that represent growth of the crystal whilst in contact with interstitial glass (e.g. sample EU30; Fig. 4). In all orthopyroxene-bearing samples, the range in plagioclase composition is much greater (An_42–96_) and zoning is common, with large compositional ranges (5–52 mol% An) within individual samples. These ranges are similar to those found in juvenile volcanic rocks of the Quill (Fig. 5b). Below An_80_ the Fe_2_O_3_ (wt%) content of plagioclase is divergent between hornblende gabbros (high-Fe) and gabbronorites (low-Fe; Fig. 5b). Plagioclase from volcanic rocks follows the gabbronorite trend (Fig. 5b). As Fe uptake by plagioclase is favoured under oxidising conditions (Sugawara 2001), the divergent trends of Fe versus An may reflect differences in magmatic redox state during differentiation, or the crystallisation of magnetite. This suggests that magnetite is important in controlling the gabbronorite trend, but less so for hornblende gabbros (Fig. 5b).

Oxides occur in more than half of samples (Fig. 3), and are present in all olivine-free assemblages (only trace amounts are present in olivine-bearing cumulates). Oxides occur as inclusions within silicate phases and interstitially, ranging in size from < 50 µm inclusions to 2 mm euhedral grains. Oxides are magnetite-rich spinels with high Al_2_O_3_ (2–9 wt%) and TiO_2_ contents (6–13 wt%) and low Cr_2_O_3_ contents (≤ 0.4 wt%). There are two compositional groups; one with higher Al# (Al/(Al + Fe^3+^+Cr) between 0.15 and 0.21 and Mg# (Mg/(Mg + Fe^2+^) between 0.15 and 0.22 and one with lower Al# (0.06 and 0.12) and Mg# (0.07 and 0.12). Oxides within ‘mushy’, non-cumulate gabbro samples (e.g. EU63 and EU95) belong to the lower Al# and Mg# group. Pleonaste spinel is present in one hornblende olivine gabbro sample EU82. These high Al_2_O_3_ spinels have previously been observed in cumulates from St. Kitts (Arculus and Wills 1980) and St. Vincent (Bouvier et al. 2008). Al-rich spinels have also been found in arc basalts where they have been interpreted to have crystallised from localised anomalously Al-rich melts from the breakdown of amphibole rich cumulates (Della-Pasqua et al. 1995).

Amphibole is present in almost all samples classified as cumulates, and is the second most modally abundant mineral. This is in contrast to Statia volcanics, where amphibole is very rare and, when present, thought to be xenocrystic from disaggregation of cumulates (Roobol and Smith 2004). Amphibole may form either euhedral crystals or, more commonly, large poikilocrysts, containing inclusions of olivine, plagioclase and clinopyroxene. Following the classification scheme of Leake et al. (2003), the majority of amphiboles are magnesio-hastingsites, with some tschermakite-pargasite in EU22 (hornblende gabbronorite), EU60 (olivine–hornblende gabbro) and EU63 (hornblende gabbro). Compositions cover a narrow range of Mg# (96–79), apart from tschermakite-pargasites in hornblende gabbronorite EU22, which have lower Mg# (83–73) (Fig. 4), similar to the range found rarely in volcanic rocks. Similarly, Al concentrations from magnesio-hastingsites are restricted (Al^IV^ 1.81–2.29 pfu), with tschermakite-pargasites in hornblende gabbronorite extending the range to lower values (Al^IV^ 1.50–1.96 pfu) (Fig. 5c). (Na + K)^A^ ranges from 0.46–0.68 in magnesio-hastingsites to 0.24–0.52 in tschermakite-pargasites. There is a positive correlation (slope = 0.52) between Al^IV^ and (Na +K)^A^ indicating that temperature-sensitive edenite exchange (Blundy and Holland 1990) is significant (Fig. 5e). Amphiboles from olivine–hornblende gabbros and hornblende gabbros cover a similar range in Al^IV^ and Al^VI^ (Fig. 5d), but amphiboles from hornblende gabbronorite extend the range to lower values that overlap those from volcanic rocks. Coupled trends in Al^IV^ and Al^VI^ are due to the pressure sensitive Al-Tschermak exchange (Johnson and Rutherford 1989) and therefore, gabbronorites may have crystallised under lower pressures. Texturally, Statia amphiboles are similar to those in other Lesser Antilles islands, however, the range in compositions is more restricted than in cumulates from Martinique, St. Kitts and Bequia (Camejo-Harry et al. 2018; Cooper et al. 2016; Melekhova et al. 2017).

**Fig. 6:**
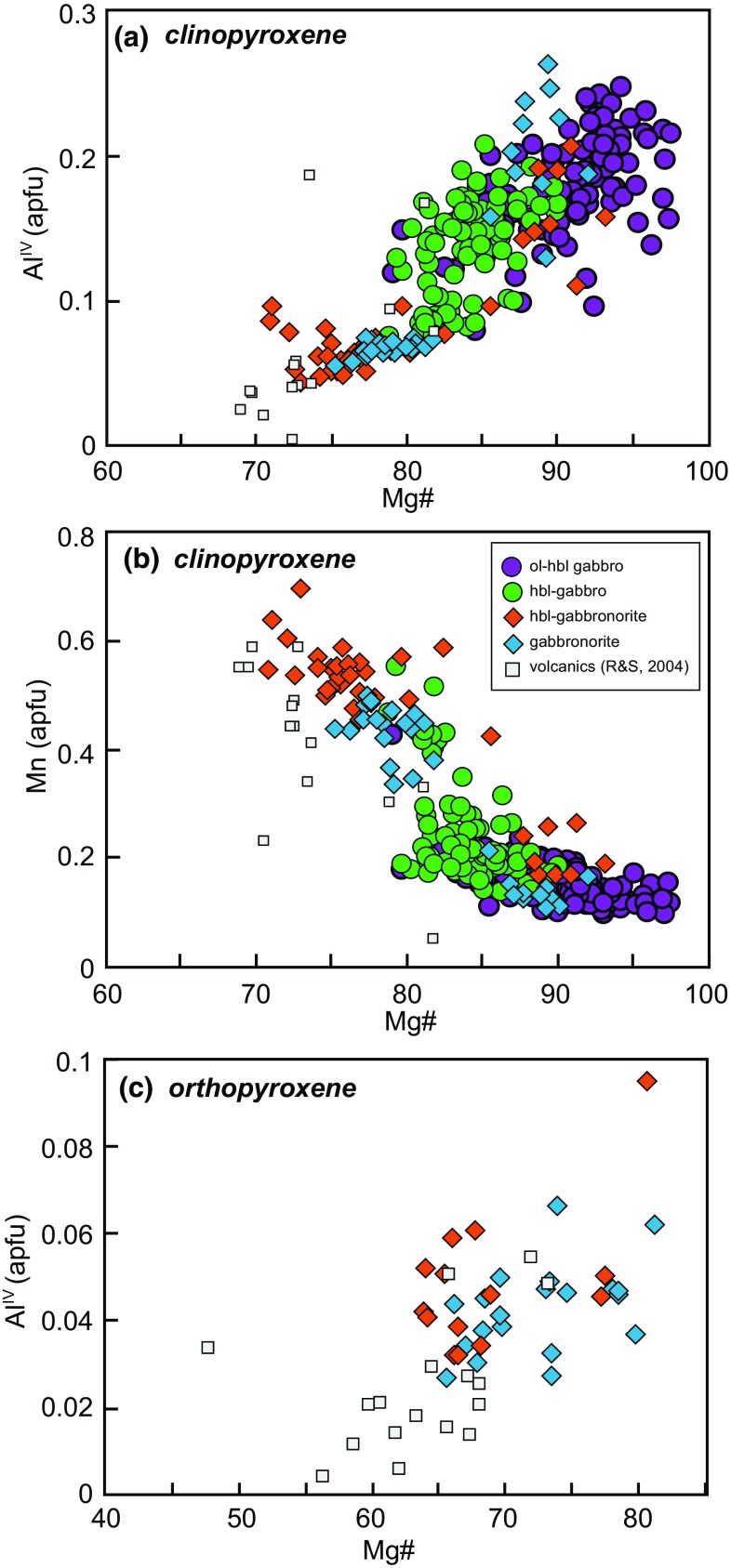
Major element pyroxene compositions from plutonic xenoliths and volcanic rocks, **a** clinopyroxene Mg# vs. Al^IV^. **b** Clinopyroxene Mg# vs. Mn (apfu). **c** Orthopyroxene Mg# vs. Al^IV^

Clinopyroxene is present in > 80% of all studied samples. Mg# spans a large range (71–98), with the lowest values in orthopyroxene-bearing samples (Fig. 4). The range in clinopyroxene composition within each sample is also relatively large (range of Mg# ≤ 14). Fe^3+^/ΣFe, estimated through stoichiometry, ranges from 0.07 to 0.89 and correlates positively with Mg#. Tetrahedral aluminium (Al^IV^) ranges from 0.04 to 0.26 and decreases with decreasing Mg# (Fig. 6a). Calcium contents (0.78–0.94 apfu) also decrease with decreasing Mg#, with the lowest Ca contents in orthopyroxene-bearing samples. Manganese (apfu) increases with decreasing Mg#, in a similar fashion to olivines (Fig. 5a), with the majority of clinopyroxene in gabbronorite samples having higher Mn, similar to the range in volcanic rocks (Fig. 6b).

Orthopyroxene is present in ~ 30% of studied samples as a subordinate phase (modal abundance < 10%). It is most commonly found in non-cumulate gabbros alongside zoned plagioclase crystals. Orthopyroxene composition ranges from En_60_ to En_76_ and Wo_1.55_ to Wo_3.86_. Minor components such as Al^IV^ (0.03–0.09 apfu) and Ca (0.03–0.08 apfu) and Ti (0.00–0.01 apfu) show little variation and no correlation with Mg# (64–81) (Fig. 6c). Orthopyroxene is present in the plutonic xenoliths from the central and northern Lesser Antilles arc (Cooper et al. 2016; Kiddle et al. 2010; Melekhova et al. 2017, 2019), but is absent from St. Vincent and Grenada in the southern Lesser Antilles arc (Melekhova et al. 2015; Stamper et al. 2014).

### Whole-rock chemistry of plutonic xenoliths and lavas

**Fig. 7:**
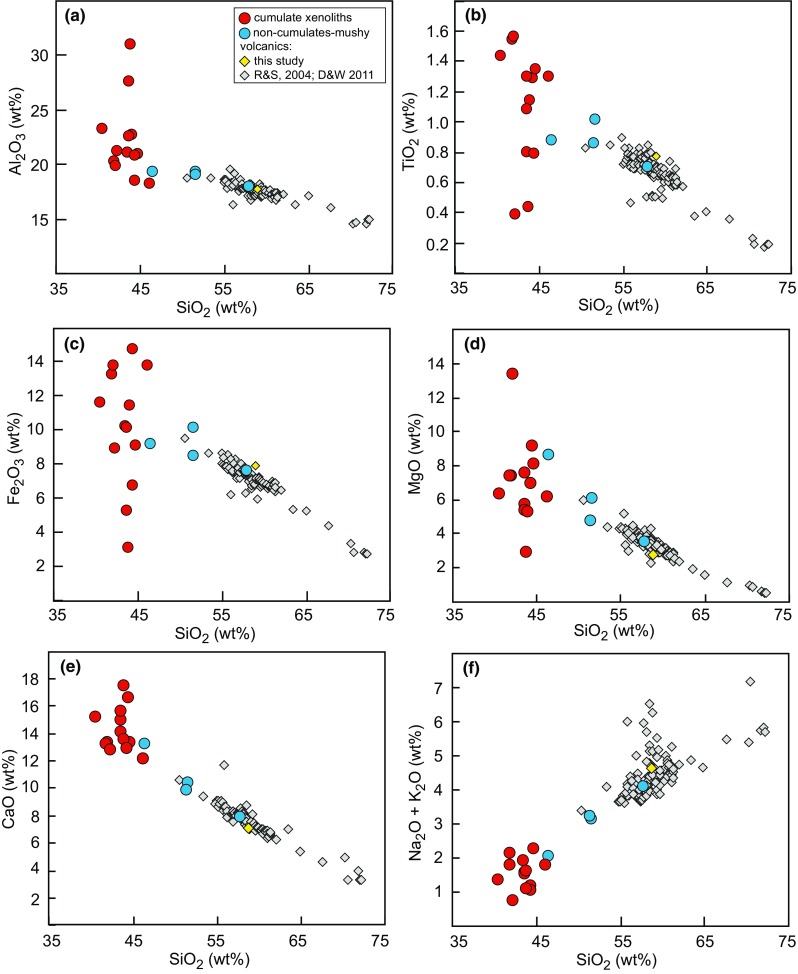
Whole-rock major element chemistry of Statia plutonic xenoliths from this study compared with volcanics (Davidson and Wilson 2011; Roobol and Smith 2004). Plutonic xenoliths have been divided into cumulates (red) and non-cumulate ‘mushy’ samples (blue)

**Fig. 8:**
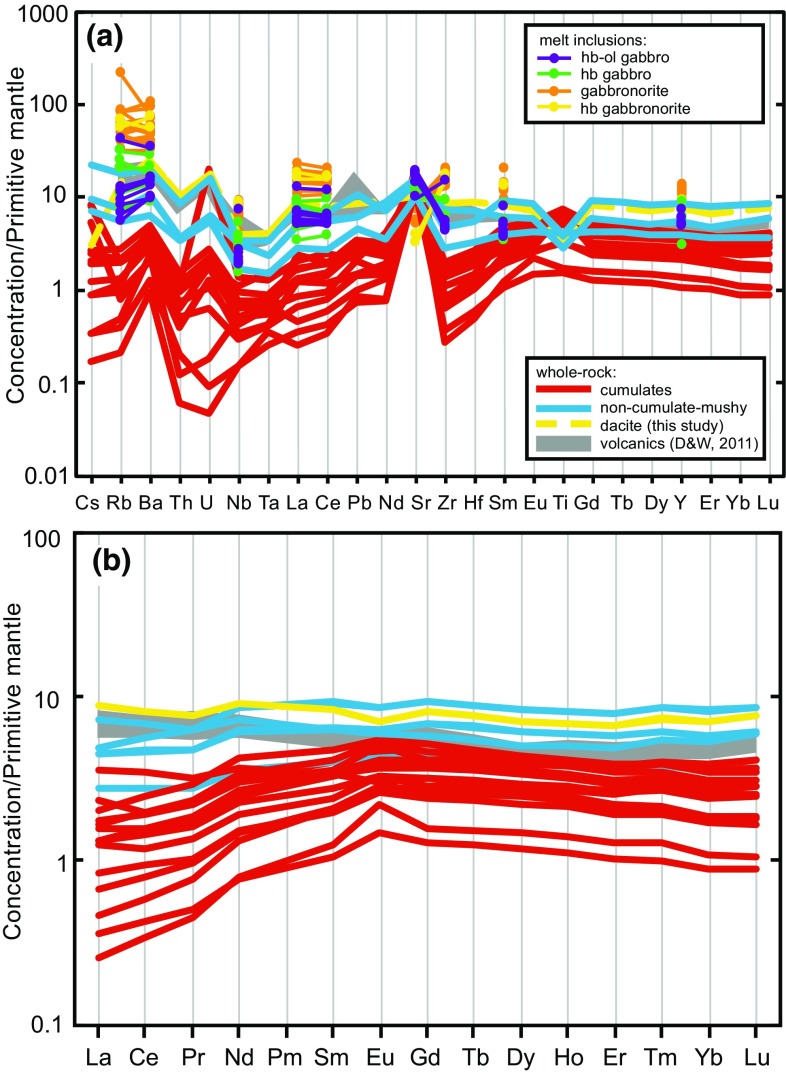
**a** Extended trace element spidergram for whole-rocks. Melt inclusions shown for comparison. **b** REE diagram of Statia plutonic xenoliths from this study, compared to volcanics (Davidson and Wilson 2011; Roobol and Smith 2004), and normalised to primitive mantle (Palme and O’Neill 2003)

Seventeen, relatively large (> 10 cm diameter) plutonic xenoliths were analysed for whole-rock major and trace element chemistry. A dacite lava was also analysed for comparison with existing volcanic geochemical data from Davidson and Wilson (2011) and Roobol and Smith (2004). Unsurprisingly, the whole-rock chemistry of the cumulate xenoliths is a direct reflection of their crystal assemblage. The majority of plutonic xenoliths lie outside the compositional field defined by lavas and do not follow any plausible liquid line of descent, consistent with a cumulate origin (Fig. 7). The cumulates have a narrow range in SiO_2_ (40.5–46.5 wt%), but a large range in MgO (3–13 wt%) and Fe_2_O_3_ (3–15 wt%). Cumulates have higher CaO (12–18 wt%) and lower Na_2_O + K_2_O (0.8–2.3 wt%) than lavas (Fig. 7). Plutonic xenoliths classified as non-cumulate, based on their ‘mushy’ texture, are geochemically distinct from cumulates and trend towards and overlie the field defined by volcanic rocks (Fig. 7). Trace element spidergrams further highlight that cumulate xenoliths are distinctive, with positive Ti anomalies, due to the abundance of amphibole in cumulate samples, and stronger positive Sr anomalies in comparison to ‘mushy’ non-cumulate gabbros and dacite, and lower LILE and HFS concentrations (Fig. 8a). U and Th are highly variable between samples, but both are in low concentrations (< 1 ppm). Cumulate xenoliths display concave-down REE profiles as they are amphibole-bearing. Those cumulates with high modal proportions of plagioclase have positive Eu (and Sr) anomalies. In contrast, ‘mushy’ non-cumulate gabbros and the dacite have higher REE abundances and flatter profiles with no or minor negative Eu anomalies (Fig. 8b).

### Major and trace element chemistry of melt

It is important to evaluate of the origin of MIs, to assess their significance as tracers of magmatic evolution. The chemistry of MIs may be modified after trapping by either crystallisation/dissolution of the host phase on the wall, crystallisation of daughter crystals (e.g. Roedder 1984) or diffusive reequilibration. Leakage of MIs may also modify the original composition of the trapped melt. Crystallisation on the walls of a MI may be difficult to observe on BSE images, particularly if the new growth is a similar composition to the host. However, the geochemistry of the analysed MIs suggest that the overall trends are produced by true variations in evolving melts, as follows: (1) MIs largely follow experimentally-determined liquid lines of lines of descent, (2) MIs from different host phases have similar major and trace element concentrations, (3) there is a consistency in MI compositions from different plutonic xenolith types, (4) MI compositions define a differentiation path consistent with the crystallisation sequence of the host xenolith. Therefore, while it is possible that minor variations in chemistry are caused by post-entrapment processes, we can have confidence that, overall, the trends in MIs represent true evolving liquids. The trends defined by MIs project back towards the plutonic xenolith whole-rock compositions, confirming that the range in chemistry is largely down to crystallisation of the phase assemblages of the plutonic xenoliths rather than addition of an exotic, unrelated melt phase.

**Fig. 9:**
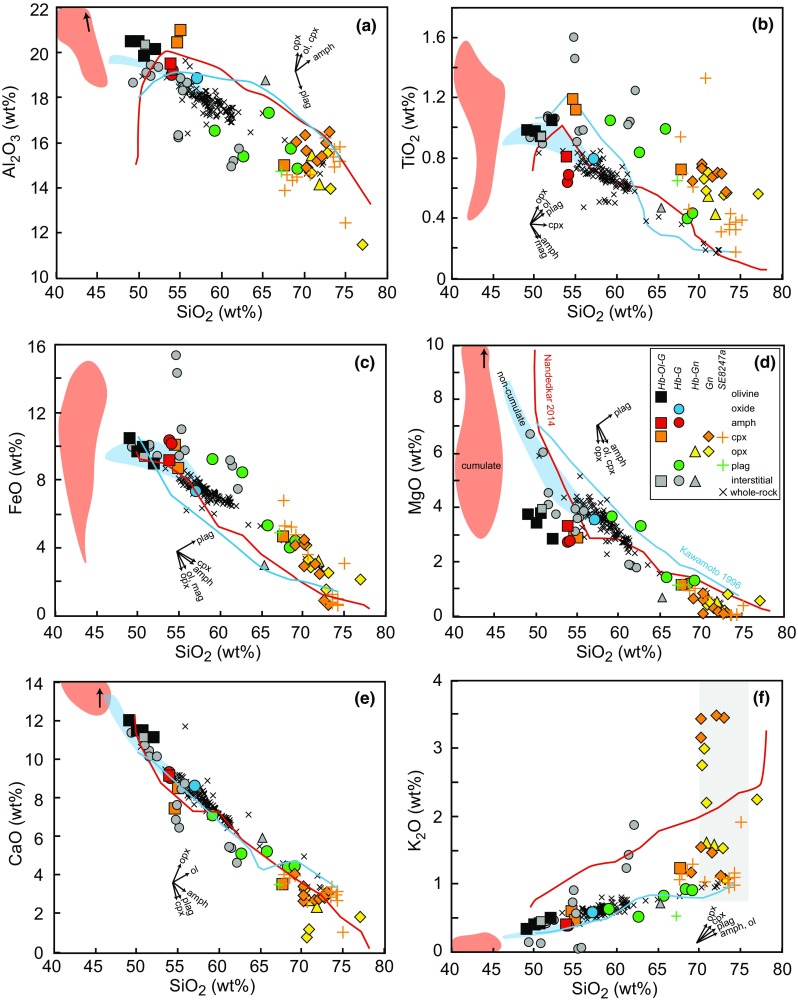
Major element chemistry of melt inclusions and interstitial glass compared to volcanic whole-rocks (Davidson and Wilson 2011; Roobol and Smith 2004) and experimental liquid lines of descent [Nandedkar et al. 2014 (red); Kawamoto 1996 (blue)]. Melt inclusions symbols are coloured based on their host phase and shapes represent different plutonic xenolith types across multiple samples. Coloured shaded areas represent plutonic xenolith whole rocks from cumulates (red) and non-cumulate gabbros (blue). Grey box in (f) encompasses the range in K_2_O and SiO_2_ in MIs from Bequia plutonic xenoliths, Bequia volcanic rocks and St. Vincent volcanic rocks (Camejo-Harry et al. 2018), and in plutonic rocks from the Peninsular Ranges Batholith (Lee and Morton 2015). Vectors indicate the effect on melt composition as a result of post-entrapment crystallisation of the labelled phases

Melt inclusions contained within Statia plutonic xenoliths have a remarkably large range in major element concentrations (Fig. 9; 49–78 wt% SiO_2_, 0.1–6.1 wt% MgO, 0.6–10.9 wt% FeO that describes an entire differentiation sequence from basalt to rhyolite. Interstitial glass has a smaller range in SiO_2_ (49–65 wt%), but similar ranges in MgO (0.1–6.7 wt%) and FeO (2.9–15.4 wt%) (Fig. 9b). Statia MIs range from basaltic-andesite to rhyolite and trends generally follow liquid lines of descent defined by experimental studies from Nandedkar et al. (2014) and Kawamoto (1996) at pressures of 0.5 and 0.7 GPa, respectively (Fig. 9). The K_2_O concentrations in Statia MIs are significantly lower than the Nandedkar et al. (2014) liquid line of descent, but are consistent with the experiments of Kawamoto (1996), which use a low K_2_O (0.27 wt%) starting material. A striking feature of the data is the large range in K_2_O at > 70 wt% SiO_2_ in MIs hosted in clinopyroxene and orthopyroxene. This inflection extends the trends defined by interstitial melts and MIs from the basaltic andesite and deviates from experimental liquid lines of descent from Nandedkar et al. (2014) and Kawamoto (1996), but follows a similar pattern to that of Nandedkar et al. (2014) at lower SiO_2_. Two plagioclase-hosted melt inclusions at 59 and 63 wt% SiO_2_ have higher FeO, MgO and TiO_2_, and lower Al_2_O_3_ than the trend defined by the other melt inclusions. Melt inclusions from a basaltic andesite sample (SE8247a) have a narrow compositional range (67–74 wt% SiO_2_) and overlie the major element chemistry of melt inclusions hosted in hornblende gabbronorites and gabbronorites. An exception to this is K_2_O which extends to higher values in high SiO_2_ plutonic xenolith MIs. Interstitial glass largely follows the same major element trends as the MIs, with the exception of melts at ~ 55 and 61–63 wt% SiO_2_ which have higher K_2_O and TiO_2_, and lower CaO and Al_2_O_3_ than other melts at the same SiO_2_ (Fig. 9). The chemistry of MIs varies systematically (and predictably) with the host phase reflecting the relative order of appearance of different minerals in the crystallisation sequence. Thus, olivine hosts the least evolved melt (~ 50 wt% SiO_2_), followed by amphibole (~ 54 wt% SiO_2_). Clinopyroxene-hosted MIs have two compositions (~ 55 and 68–73 wt% SiO_2_) straddling plagioclase MIs (59–69 wt% SiO_2_). The clinopyroxene-hosted MIs show a correlation with the host crystal, with MIs containing ~ 55 wt% SiO_2_ hosted in higher Mg# and Al^IV^ clinopyroxene from olivine hornblende gabbros, compared with MIs at 68–73 wt% SiO_2_ from gabbronorites (Fig. 6). Orthopyroxene-hosted MIs are the most evolved (71–77 wt% SiO_2_). Plagioclase-hosted MIs are more evolved than amphibole MIs in hornblende gabbros, which does not reflect the earlier appearance of plagioclase. Therefore, it is likely that the plagioclase MIs were trapped during the later stages of growth, once amphibole had begun to crystallise. In contrast to the MI compositional array, volcanic whole rock major element chemistry defines straight line trends between 50 and 72 wt% SiO_2_.

**Fig. 10:**
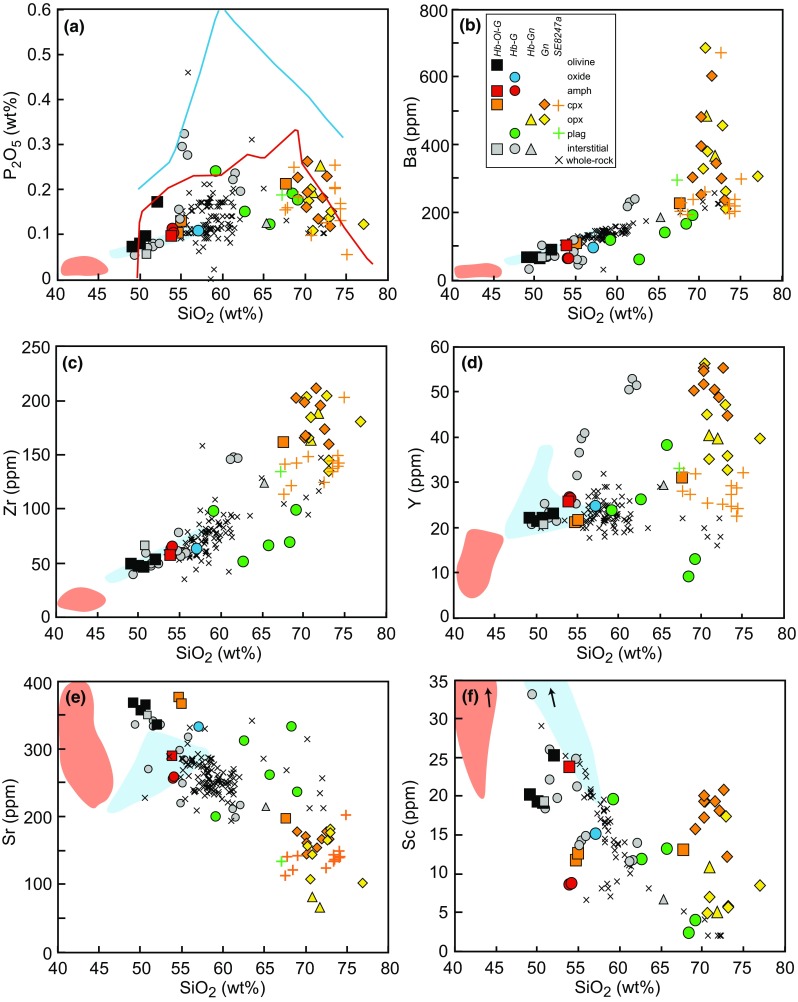
Selected minor and trace element chemistry of melt inclusions and interstitial glass compared to volcanic whole-rocks (Davidson and Wilson 2011; Roobol and Smith 2004). Colours denote host phase and shapes represent different plutonic xenolith types. Coloured shaded areas represent plutonic xenolith whole rocks from cumulates (red) and non-cumulate gabbros (blue). Lines in (a) represent experimental liquid lines of descent (Nandedkar et al. 2014 (red); Kawamoto 1996 (blue))

Minor and trace element MI concentrations also display considerable variation (Fig. 8a), including typical fractionation trends with SiO_2_ wt% (Fig. 9). P_2_O_5_ increases in MIs up to an inflection at ~ 65 wt% SiO_2_, then decreases in melt inclusions from hornblende gabbronorites, gabbronorites and the basaltic andesite sample (Fig. 10a). The high-SiO_2_ MIs which display a threefold increase in K_2_O (Fig. 9f) also show enrichments in incompatible trace elements (Fig. 8a) and clear inflections in Ba, Zr, Y, Cl and Sc (Fig. 10). Ba in MIs (32–686 ppm) increase with increasing SiO_2_ wt%, with a prominent inflection and threefold increase in Ba at SiO_2_ > 70 wt% (Fig. 10b). Overall, Zr (39–211 ppm) continues to increase over the full range of SiO_2_, suggesting that zircon saturation has not been reached (Fig. 10c). Sr in MIs (72–377 ppm) and in interstitial glass (200–351 ppm) decrease with increasing SiO_2_ wt% (Fig. 10e). Plagioclase-hosted MIs from hornblende gabbros containing between 60 and 70 wt% SiO_2_ overlie a population of volcanic whole rock analyses. Y increases steadily until ~ 65 wt% SiO_2_, then has a sharp two-fold increase in MIs from hornblende gabbronorite and gabbronorite (Fig. 10d). The interstitial melts with high K_2_O at ~ 55 and 61–63 wt% SiO_2_ diverge to higher Ba, Zr and Y and Cl than the general MI trends (Fig. 10). This inflection in Y in interstitial melts is mirrored by whole rock concentrations of non-cumulate ‘mushy’ samples. Sc decreases in MIs and interstitial melt until ~ 65 wt% SiO_2_, after which it sharply increases threefold (Fig. 10f). Unlike the trends shown by MIs, the volcanic whole rock trace element chemistry defines either straight line trends as seen in major elements, or no trends at all.

### Volatile content of melt inclusions

**Fig. 11:**
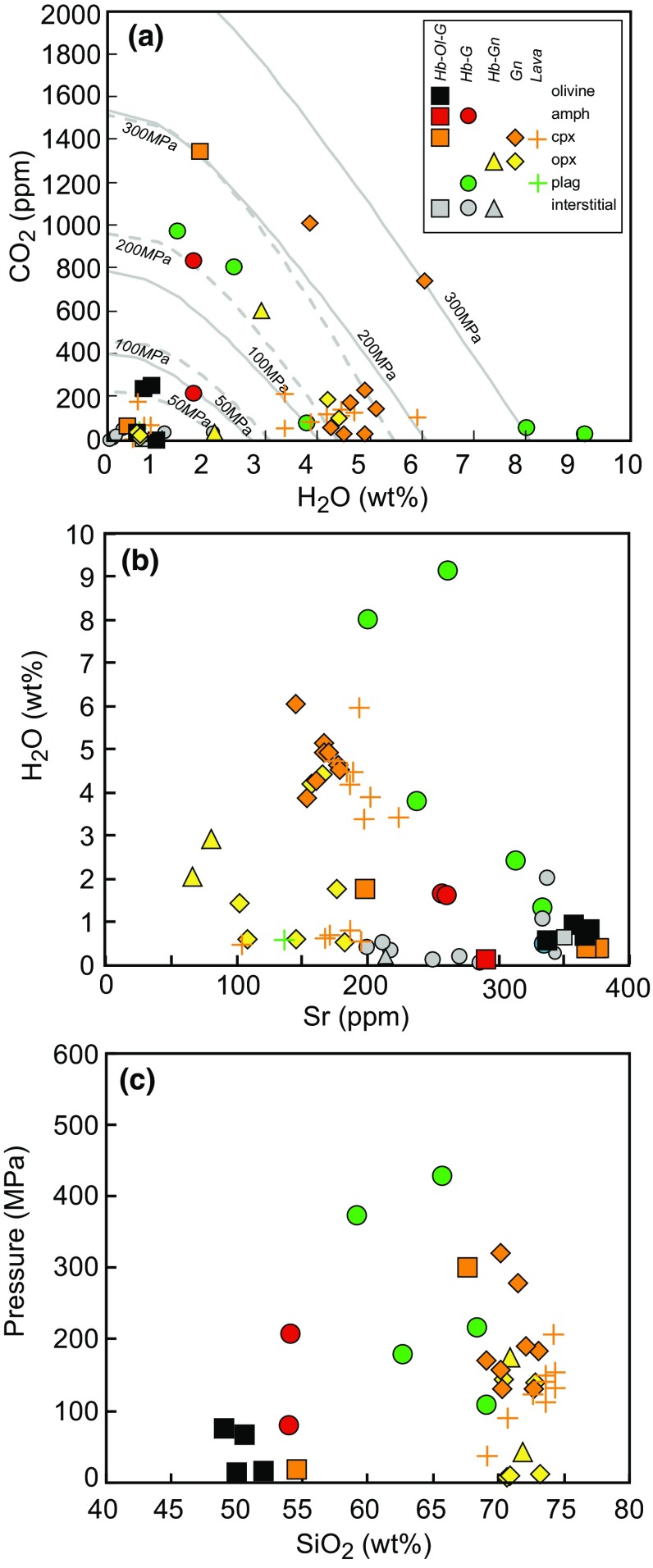
**a** H_2_O wt% versus CO_2_ (ppm) in Statia MIs. Isobars were drawn using VolatileCalc for generic basalt at 1000 °C (dashed grey line) and generic rhyolite at 800 °C (solid grey line). One value (3737 ppm CO_2_, 0.36 wt% H_2_O) is not shown to enhance clarity of the dataset. **b** Strontium versus H_2_O of Statia MI’s. The trend to higher H_2_O wt% at ~ 200 ppm suggests this population of MIs are water-saturated. **c** SiO_2_ versus estimated volatile saturation pressures using MagmaSat (Ghiorso and Gualda 2015)

Melt inclusions from Statia cover a large range in H_2_O (0.1–9.1 wt%), although ~ 50% of MIs have H_2_O contents < 2 wt% (Fig. 11). Plagioclase-hosted MIs from hornblende gabbro samples EU28 (8.0 wt%) and EU63 (9.1 wt%) have the highest H_2_O contents. All interstitial glasses analysed have H_2_O contents < 2.5 wt%, with most recording < 1 wt%. CO_2_ contents of Statia MIs range from 0 to 1350 ppm, with 57% below 100 ppm (Fig. 11a). On plots of H_2_O versus CO_2_ contents (Fig. 11a), MIs from different plutonic xenolith types do not show a clear trend, however, there is a group of MIs from hornblende gabbronorites, gabbronorites and the basaltic andesite sample at 3–6 wt%, H_2_O < 300 ppm CO_2_. These samples lie between the 100 and 200 MPa vapour saturation isobars, calculated using VolatileCalc (Newman and Lowenstern, 2002) for a rhyolite at 800 °C. These MIs are from a population that shows an increase in H_2_O with decreasing Sr, suggesting they are water-saturated (Fig. 11b). However, a significant number of other MIs have low H_2_O (< 1 wt%) at < 200 ppm Sr, suggesting they are not water-saturated or have lost water. The highest water contents are found at intermediate SiO_2_ contents (Fig. 11). A decrease in CO_2_ with increasing concentrations of incompatible trace elements would confirm the melt inclusions were volatile-saturated (e.g. Wallace et al. 1999). However, no clear trends are observed when CO_2_ is plotted against major or trace element concentrations. Therefore, we suggest that a proportion of the analysed MIs are volatile-undersaturated.

In addition to H_2_O and CO_2_, volatile species (Li, B, F, Cl) were analysed. Li contents of Statia MIs range from 0 to 46 ppm and are positively correlated with K_2_O and B, but do not show clear trends with other measured volatiles. Cl concentrations are highly enriched in Statia MIs (≤ 0.4 wt%) and increase with increasing SiO_2_, with a large range above 65 wt% SiO_2_ (0.15–0.4 wt% Cl) in hornblende gabbronorites and gabbronorites (Fig. 10f). Cl contents generally increase with increasing K_2_O and other volatiles (H_2_O, F, B). B contents of Statia MIs range from 1 to 24 ppm and have positive correlations with K_2_O and H_2_O.

## Discussion

### Intensive variables within the Statia plumbing system

Determining the intensive variables (*P*–*T*–H_2_O) from which plutonic xenoliths from Statia were formed is vital in understanding the storage conditions of the sub-volcanic plumbing system, as well as the evolution of magmas within the arc crust. Here, we integrate several different barometry and thermometry approaches to provide a robust estimate of crustal storage conditions for Statia plutonic xenoliths.

#### Volatile contents and saturation pressures

The H_2_O and CO_2_ contents of MIs were used to calculate volatile saturation pressures using the method of Ghiorso and Gualda (2015). A number of the analysed MIs contain a vapour bubble. These bubbles may contain substantial concentrations of CO_2_ which would result in underestimates of the original dissolved CO_2_ (Moore et al. 2015). As discussed above, a significant number of analysed MIs are likely to be water undersaturated and/or may have undergone some volatile loss and reequilibration (Fig. 11b). We, therefore, consider the majority of pressure estimates as minima. Volatile saturation pressures, calculated using the individual chemistry of each MI at a fixed (950 °C) temperature, cover a large range 0–425 MPa (Fig. 11) suggesting crystallisation and/or storage in the upper-middle crust (0–15 km).

The high water contents recorded in Statia MIs (0.1–9.1 wt% H_2_O) are consistent with high water contents measured in melt inclusions from Montserrat (Humphreys et al. 2009; Cassidy et al. 2015), Bequia (Camejo-Harry et al. 2018) and Dominica (Balcone-Boissard et al. 2018) up to 6.2, 7.8 and 8 wt% H_2_O, respectively. In addition, independent estimates of melt water contents using orthopyroxene in andesites from Soufrière Hills Volcano, Montserrat, suggest melt water concentrations of 6–9 wt% H_2_O (Edmonds et al. 2016). The high water contents in Statia plutonic xenoliths may account for the presence of the highly calcic plagioclase (An_86–99_ in cumulates and An_42–96_ in orthopyroxene-bearing, non-cumulate gabbros) that crystallised alongside relatively low Fo (Mg# 69–80) olivine. This association is common in Lesser Antilles plutonic xenoliths (Arculus and Wills 1980; Cooper et al. 2016; Melekhova et al. 2015; Stamper et al. 2014; Tollan et al. 2012) and has been shown experimentally to be the result of high Ca/Na melt compositions of primitive melts and/or high water contents (9–13 wt% H_2_O for St. Kitts lavas and xenoliths, Melekhova et al. 2017). The relatively evolved olivine compositions suggest that the plutonic xenoliths have crystallised from melts that have undergone prior differentiation of mafic minerals, including olivine. Therefore, although not represented in the xenolith record, we expect the lower crust beneath Statia, at depths greater than those recorded by the xenoliths, to be composed of olivine ± clinopyroxene-rich cumulates derived from primary hydrous basaltic melts. An additional indication for high water contents and the suppression of plagioclase is the maximum Al_2_O_3_ concentration of the melt inclusions (~ 21 wt%, Fig. 9a) according to the melt-based hygrometer of Pichavant and Macdonald (2007), which would suggest ~ 6 wt% dissolved H_2_O.

#### Multiple reaction barometry

Ziberna et al. (2017) established a multiple-reaction approach that uses a least-squares minimization to average the pressures from individual mineral equilibria. This method can be applied to plutonic xenoliths from Statia with the assemblage clinopyroxene + olivine + plagioclase equilibria (COlP). Pressures were calculated for 10 plutonic xenolith samples using mineral analyses from adjacent grains (Table 1 and Online Resource 1). Pressures range from 0.9 to 3.7 kbar, covering a very similar range to the volatile saturation pressures. Uncertainties on the COlP estimates are large (1.2–4.7 kbar), but estimates from samples with the same assemblage are consistent. No xenoliths contained the assemblage spinel + clinopyroxene + olivine + plagioclase assemblage suitable for the more precise Ziberna et al. (2017) SCOlP barometer.

**Table 1:** Summary of geothermobarometry estimates

		Zircon sat T (°C)	Apatite sat T (°C)	Two-px T (°C)	COlP (kbar)	MIs (kbar)
*hbl*-*ol gabbro*	EU84	690	625			0.1–0.7
*hbl*-*ol gabbro*	EU49	750	770		2.09 (3.28)	0.1–3.0
*hbl*-*ol gabbro*	EU77				3.69 (3.88)	
*hbl*-*ol gabbro*	EU59				2.08 (4.23)	
*hbl*-*ol gabbro*	EU16				3.5 (1.65)	
*hbl*-*ol gabbro*	EU82				2.54 (4.7)	
*hbl*-*ol gabbro*	EU93				2.55 (4.14)	
*hbl*-*ol gabbro*	EU1				2.95 (3.15)	
*hb gabbro*	EU28	745	770			0.8–3.7
*hb gabbro*	EU63	765	915			1.1–4.3
*hb gabbro*	EU23	740	760			
*hb gabbro*	EU12	755	760	880	2.33 (1.18)	
*hb gabbronorite*	EU22	810	875	920		
*hb gabbronorite*	EU60			915		
*gabbronorite*	EU18			910	0.91 (2.45)	
*gabbronorite*	EU65	855	1005	930		0.4–1.7
*gabbronorite*	EU68			945		
*gabbronorite*	EU95	870	975	945		1.3–3.2
*gabbronorite*	EU2	845	970			< 0.1

Zircon (Watson and Harrison 1983) and apatite (Harrison and Watson 1984) saturation temperatures and two-pyroxene temperatures (Putirka 2008) are averages from each plutonic xenolith. Multiple reaction barometry (COlP) uses method of Ziberna et al. (2017). Uncertainties on COIP estimates are shown in brackets. Melt inclusion saturation pressures calculated using MagmaSat (Ghiorso and Gualda 2015)

#### Temperature estimates

Apatite and zircon saturation temperatures were calculated following methods of Harrison and Watson (1984) and Watson and Harrison (1983) respectively (Table 1). Apatite saturation temperatures range from 625 to 1005 °C. In the 4 samples inferred to have saturated in apatite (Fig. 10a; EU2, EU63, EU65, EU95), temperatures span a smaller range from 915 to 1005 °C. The lower SiO_2_ samples where apatite saturation has not been reached (Fig. 10a) therefore, represent approximate minimum temperatures. Zircon saturation estimates range from 690 to 870 °C and represent approximate minimum temperatures as zircon concentrations of plutonic xenolith melts suggest that zircon saturation was not reached (Fig. 10c). This is further demonstrated by the offset between zircon saturation temperatures and apatite saturation temperatures (105–150 °C) in the 4 samples inferred to have saturated in apatite (Table 1; Fig. 10a). Two-pyroxene temperatures were also calculated using Eq. 37 of Putirka (2008) for samples containing both clinopyroxene and orthopyroxene (Table 1). Equilibrium of pyroxene pairs was assessed using the *K*_D_ (Fe–Mg) and average temperatures, taken from the appropriate pairs, range from 880 to 945 °C. The temperature range within each sample varies considerably between 22 and 220 °C and reflects the compositional range of pyroxenes (e.g. EU18). Uncertainties (standard error of estimate) based on the regression of experimental data are ± 60 °C (Putirka 2008).

#### Experimental petrology

**Fig. 12:**
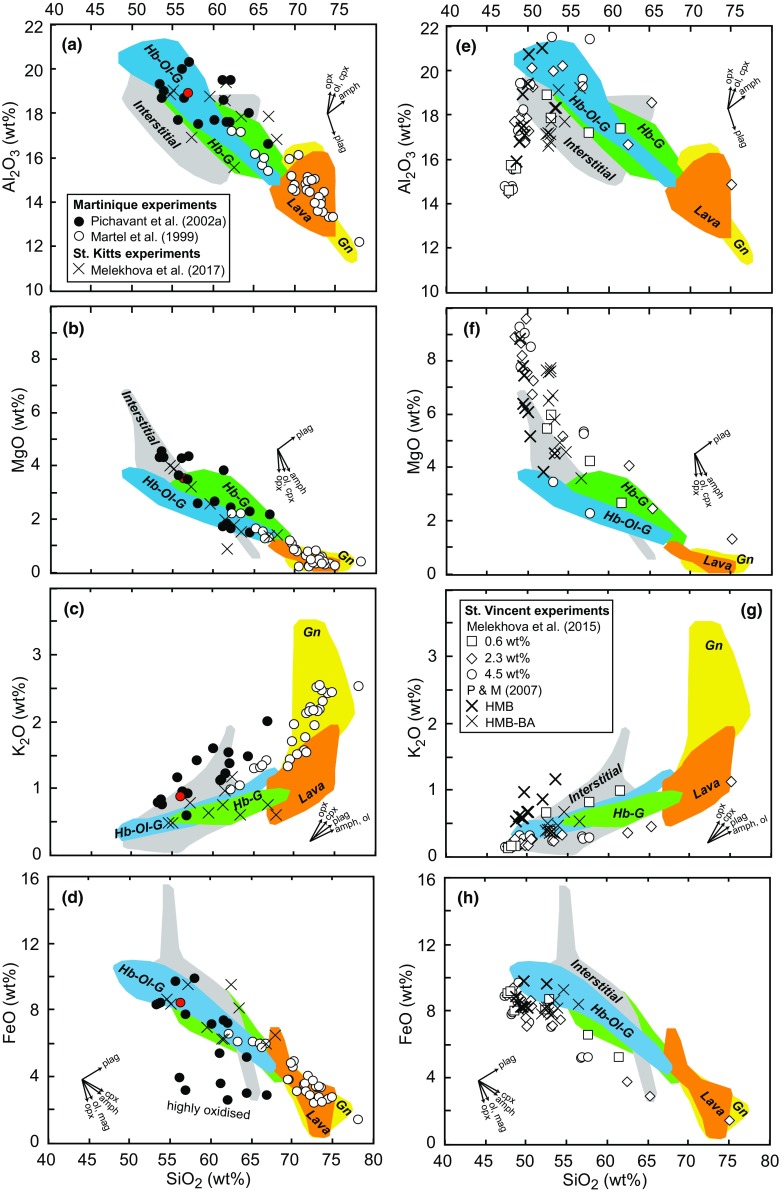
Comparison of melts produced in Lesser Antilles experiments with Statia melt inclusions from each plutonic xenolith type. Coloured fields represent the range of MI compositions from each plutonic xenolith type as shown in Fig. 9 and symbols represent experimental melt compositions (normalised to 100%, volatile free). Experiments from Martinique and St. Kitts are in **a**–**d**. Red circle indicates an olivine-bearing assemblage from Pichavant et al. (2002a). Experiments from St. Vincent are shown in **e**–**h**. Vectors indicate the effect on MI composition as a result of post-entrapment crystallisation of the labelled phases

The melt inclusion compositions recorded in Statia plutonic xenoliths cover a similar range to the liquid lines of descent defined by experimental melts from the Lesser Antilles (Fig. 12; Martel et al. 1999; Melekhova et al. 2015, 2017; Pichavant and Macdonald 2007; Pichavant et al. 2002a) and from experiments with similar starting compositions (Fig. 9; Kawamoto 1996; Nandedkar et al. 2014). The general agreement of the experimental and natural melts implies that fractional crystallisation plays a significant role in magma genesis at Statia. We can use the run conditions (temperature, pressure, water contents and ƒO_2_) of these experiments to explore the controls on the large range of Statia MI compositions. The orthopyroxene-bearing Martinique andesite experiments of Martel et al. (1999) overlap the gabbronorite and lava sample MI compositions (Fig. 12). The temperatures of these experiments range from 876 to 925°C and were run under hydrous conditions (~ 4–7 wt%). These high water contents are consistent with the range of water contents measured in gabbronorite MIs (50% between 4.2 and 6.1 wt%). The experimental pressures (2–3 kbar) suggest the gabbronorites and lavas crystallised in the upper crust. Experimental melts from St. Kitts (Melekhova et al. 2017) are closest in composition to MIs from hornblende gabbros (Fig. 12). Experiments with appropriate hornblende-bearing assemblages were run at higher temperatures (950–975°C) than those of Martel et al. (1999), but at similar pressures (2.4 kbar). Only experiments run at *X*H2O of 1.0 and 0.66 (3.6–10 wt%), and not *X*H2O = 0.33 produced hornblende in the crystallising assemblage. One Martinique experiment of Pichavant et al. (2002a) run at 1000°C, 4.2 kbar and 6.8 wt% H_2_O (2002a) contained olivine and amphibole, appropriate for comparison with hornblende olivine gabbro assemblages. The experimental melt composition lies within the range of Al_2_O_3_ and FeO of MI’s from hornblende olivine gabbros, but has higher MgO and K_2_O. Two experiments on St. Vincent basalt with 4.5 wt% H_2_O by Melekhova et al. (2015), run at 1030–1100°C and 7–10 kbar, produced a hornblende olivine gabbro assemblage with melt Al_2_O_3_ and K_2_O contents in the range of Statia MI. The experimental melts have higher MgO and lower FeO, which are likely the consequence of being run under relatively oxidising conditions (see discussion below). The St. Vincent experimental melts of Pichavant and Macdonald (2007) overlie overly the low SiO_2_ end of the Statia MI compositional array. However, no experimental assemblages which crystallised both olivine and hornblende were recorded.

No coexisting magnetite and ilmenite pairs are found in the Statia plutonic xenoliths, therefore, ƒO_2_ cannot be estimated. Experimental studies show that ƒO_2_ strongly affects the FeO content of the melt (Fig. 12). Experiments from Martinique (Pichavant et al. 2002a) and St. Vincent (Melekhova et al. 2015) run at highly oxidising conditions (> 3 log units above NNO) produced melts with lower FeO trends that fall outside the range of Statia MIs (Fig. 12d, h). Experimental melt that do overlie the FeO concentrations of Staita MIs were performed between NNO and NNO +1 (Martel et al. 1999; Melekhova et al. 2017; Pichavant et al. 2002a; Pichavant and Macdonald 2007). Therefore, we estimate that redox conditions of crystallisation of Statia plutonic xenoliths lay in this range.

Overall, the experiments suggest that the Statia cumulates and associated melt compositions can be produced under hydrous and mildly oxidising conditions. Temperature appears to have a control on the varying plutonic xenolith assemblages, from > 1000°C in hornblende-olivine gabbros, to 950–975°C for hornblende gabbros, to 876–925°C in orthopyroxene-bearing assemblages. Hornblende-gabbros and gabbronorites were likely stored in the upper crust at pressures 2–3 kbar, whereas olivine-bearing assemblages likely crystallised deeper at ≥ 4 kbar.

### The record of magma differentiation at Statia

Statia plutonic xenoliths provide evidence for magmatic differentiation processes including fractional crystallisation, mixing and amphibole breakdown that took place over a range of depths. Melt inclusions have a large range in major element concentrations that define a differentiation trend from basalt to rhyolite (Fig. 9). Major element MI trends generally follow liquid lines of descent of experimental melts (Fig. 9) from Nandedkar et al. (2014) and Kawamoto (1996). These trends project back to plutonic xenolith whole-rock compositions indicating that chemistry of the MIs is largely driven by crystallisation of the plutonic xenolith phases. Statia MIs have low K_2_O concentrations between 50 and 65 wt% SiO_2_, inconsistent with the liquid line of descent of Nandedkar et al. (2014). This low-K_2_O trend is also distinct from other Lesser Antilles MIs (Pichavant et al. 2002a, b; Melekhova et al. 2015; Humphreys et al. 2010) that typically follow a higher K_2_O path. The low-K_2_O MIs are likely a consequence of a low-K_2_O primitive magma (analogous to the starting material of Kawamoto (1996) experiments) and crystallisation under high water contents (Müntener and Ulmer, 2018; Erdmann and Koepke, 2016). K_2_O enrichment in residual melts is largely a function of the amount of crystallisation, as K is an incompatible element in the absence of biotite. High H_2_O contents have been shown experimentally to suppress K_2_O enrichment with increasing SiO_2_ in the melt largely due to the increased amount of amphibole crystallisation that, in turn, serves to increase the rate of SiO_2_ enrichment relative to dry conditions where pyroxenes and plagioclase crystallisation dominate (Müntener and Ulmer 2018; Erdmann and Koepke 2016). The contrast between ‘dry’ and ‘wet’ conditions on K_2_O concentrations can be seen in experimental melts from St. Vincent (Fig. 12g; Melekhova et al. 2015). Moreover, high H_2_O contents are consistent with the observed water contents of Statia MIs (up to 9 wt%).

Melt inclusions from hornblende gabbronorite and gabbronorite show an inflection in P_2_O_5_ variations that indicates apatite saturation (Fig. 10). The inflection occurs at ~ 65 wt% SiO_2_, and is similar to the liquid line of descent from experiments of Nandedkar et al. (2014). This trend is consistent with the presence of accessory apatite in a number of gabbronorite samples. Unlike P, the Zr concentrations of Statia melts do not show an inflection with increasing melt SiO_2_ (Fig. 10) suggesting that the melts largely remain zircon undersaturated. This is due to the fact that zircon typically saturates at higher SiO_2_ and lower temperature than apatite. A melt between 60 and 70 wt% SiO_2_, with 0.1–0.25 wt% P_2_O_5_ will saturate apatite at 900 °C (Harrison and Watson, 1984), 25–200 °C higher than the temperature of zircon saturation over the measured range of MI Zr concentrations (50–200 ppm) (Watson and Harrison 1983).

Two plagioclase-hosted MIs from hornblende gabbros have higher FeO, MgO and TiO_2_ than the other MI compositional array (Fig. 9). Plagioclase in hornblende gabbros also has a trend to high Fe and these samples are rich in Fe–Ti oxides (3–11%; Fig. 2). MIs hosted in plagioclase from hornblende gabbro also contain the highest water contents (Fig. 11) and therefore, may have also crystallised under higher ƒO_2_ conditions that would enhance the crystallisation of magnetite as well as increasing the partitioning of Fe into plagioclase. However, if this was the case then the FeO content of the melt would be expected to decrease, as shown by experiments run at highly oxidising conditions (Fig. 12). As this is not the case for Statia MIs, it is possible that the two plagioclase-hosted MIs have been affected by post entrapment crystallisation. Crystallisation of plagioclase on the MI wall would increase FeO, MgO and TiO_2_ and decrease Al_2_O_3_ of the melt. This effect is shown by the vectors in Fig. 9. Crystallisation of magnetite in these samples at melt compositions ~ 58–62 wt% SiO_2_, could promote the small compositional gap in MIs at 60–65 wt% SiO_2_ (discussed further below) by rapidly increasing the SiO2 concentration of the residual melt (Grove and Donnelly-Nolan, 1986).

Plutonic xenolith-hosted MIs > 65 wt% SiO_2_ from gabbronorites and hornblende gabbronorites display a significant range in incompatible elements (K_2_O, Ba, Y, Cl in Figs. 9 and 10). These sharp increases in element concentrations are not seen in Lesser Antilles experiments (Fig. 12) or the experiments of Nandedkar et al. (2014) and Kawamoto (1996) (Figs. 9, 10). This trend appears to be common in the Lesser Antilles with similar increases in K_2_O over narrow ranges in SiO_2_ and MgO in MIs from Kick-‘em-Jenny (Camejo-Harry et al. 2019) and in both lavas and plutonic xenolith hosted MIs from Bequia (Camejo-Harry et al. 2018), shown by the grey field in Fig. 9f. There are a number of processes which may account for the range in incompatible elements in the evolved MIs. Firstly, amphibole breakdown reactions (e.g. Buckley et al. 2006; Rutherford and Hill, 1993) may release into the melt elements such as K_2_O, Ba, Y (Figs. 8, 9) that are relatively enriched in amphibole relative to product anhydrous minerals, such as plagioclase, oxides and pyroxenes. Roobol and Smith (2004) observed that amphiboles in volcanic products of the Quill commonly contain a reaction rim of magnetite and pyroxene, or may be completely replaced, and glomerocrysts with assemblages of plagioclase, pyroxene and magnetite are present. Amphibole breakdown within the plutonic xenoliths themselves is supported by melt Sc concentrations (Fig. 10) that decrease in in amphibole-bearing samples between ~ 50 and 70 wt% SiO_2_, but sharply rise in MIs from amphibole-free gabbronorites > 70 wt% SiO_2_. Sc is compatible within amphibole (and clinopyroxene to a lesser extent). The direct evidence for amphibole breakdown reactions implies that gabbronorite xenoliths and lavas were stored outside of the amphibole stability field (temperature, pressure and H_2_O) for some time prior to eruption. However, even complete breakdown of amphibole could not release enough K_2_O into the melt for the observed threefold increase in MI compositions and so an additional process is needed.

Secondly, the gabbronorites may represent a separate, less hydrous fractionation series. A ‘dry’ fractionation trend could produce gabbronorites without prior amphibole crystallisation and at higher pressures than a ‘wet’ fractionating sequence. These ‘dry’ trends have been observed to crystallise gabbronorite assemblages in crustal sections of the Kohistan arc (Jagoutz et al. 2011), and their low H_2_O could also account for a sharp rise in K_2_O as shown by anhydrous experiments of Müntener and Ulmer (2018). However, Statia gabbronorite hosted MIs contain up to 6 wt% water and Lesser Antilles experiments produce orthopyroxene under hydrous conditions (e.g. Martel et al. 1999), suggesting more hydrous conditions during fractionation.

Thirdly, it is possible that fractional crystallisation alone drove melts to very high K_2_O. In this scenario, a eutectic composition is reached and quartz + feldspar crystallisation (either as cumulate phases or late stage microlites) would increase the incompatible element concentration of the melt, whilst buffering the melt SiO_2_ concentration. Similar enrichments (threefold increase in K_2_O) in incompatible elements at evolved compositions > 71 wt% SiO_2_ (grey field in Fig. 9f) have been observed in whole-rocks from the plutonic rocks of the Peninsular Ranges Batholith (Lee and Morton 2015). In this case, the enriched compositions are inferred to be due to the crystallisation of evolved cumulates. However, there is no direct evidence for quartz crystallisation (either as cumulate phases or late stage microlites) in plutonic xenoliths from Statia (only present in one volcanic sample from Roobol and Smith 2004). Quartz is found in Lesser Antilles plutonic xenoliths from Saba, St. Kitts and St. Lucia (Arculus and Wills 1980), so it may be possible that this assemblage is present in the Statia magmatic system but has not been erupted, leaving a cryptic crystallisation signature.

Finally, potassium may be added to the system from an external source, such as the percolation of a chemically distinct evolved melts or fluids. This scenario has been shown to cause the breakdown of cpx and subsequent crystallisation of chemically variable amphibole in cumulates from Martinique (Cooper et al. 2016). Melting of additional cumulate phases, particularly biotite which is enriched in incompatible elements (Fig. 8a; Beard et al. 2005; Reubi and Blundy 2008) is a potential source of these distinct melts. This process has been proposed to explain high-K ‘exotic’ melt inclusions from Volcán de Colima, Mexico, that are trapped during partial melting of cumulates during assimilation in a silicic melt (Reubi and Blundy 2008). Although no biotite-bearing samples are found on Statia, biotite is present in plutonic xenoliths from St. Kitts, St. Lucia and Bequia (Arculus and Wills 1980; Camejo-Harry et al. 2018). At Statia, the extent of enrichment in incompatible elements in the evolved MIs are variable (Figs. 9, 10), which cannot be accounted for by a single process. Therefore, we suggest that a combination of an amphibole breakdown reaction alongside the open-system percolation of chemically distinct evolved melts can account for this geochemical variability.

### The record of magma mixing at Statia

The MIs display an apparent andesitic compositional gap (only one MI between 60 and 65 wt% SiO_2_; Online Resource 3), which is a common worldwide feature of volcanic rock suites (Brophy 1991; Daly 1925) and melt inclusions (Reubi and Blundy 2009). The origin of the ‘Daly gap’ has been attributed to various processes, including; partial melting of the crust (Chayes 1963), crystal fractionation and magma chamber processes (Brophy 1991; Grove et al. 1997; Marsh 1981), melt extraction from crystal mushes (Dufek and Bachmann 2010), silicate melt immiscibility (Charlier et al. 2011), variations in the heat and water content of the magmatic system (Melekhova et al. 2013) and early crystallisation of SiO_2_-poor phases such as magnetite and amphibole (Grove and Donnelly-Nolan 1986). In contrast to the MIs, many interstitial melts and volcanic whole-rocks have compositions between 60 and 65 wt% SiO_2_ (Online Resource 3). The volcanic whole-rocks and interstitial melts provide geochemical evidence for the mixing of a diversity of magmas (liquids and crystalline components). The volcanic products erupted from the Quill (Statia) have a similar range in compositions (whole-rock values of 51–73 wt% SiO_2_) to the MIs analysed in this study. In contrast to the curved differentiation trends defined by the MIs, however, whole-rock chemistry displays straight-line geochemical trends connecting the least and most evolved MI compositions (excluding the high K_2_O, Ba, Y MIs). The presence of rhyolitic and basaltic melt inclusions, but dominantly andesitic whole-rocks with straight-line geochemical trends, suggest that the final erupted magmas at Statia could be mixtures of a diversity of magmas from basaltic to rhyolitic in composition (Reubi and Blundy 2009). Interstitial melts in the plutonic xenoliths provide evidence for additional mixing processes. A number of interstitial melts with high K_2_O at ~ 55 and 61–63 wt% SiO_2_ diverge to higher Ba, Zr and Y and Cl than the general MI trends (Fig. 10). These melt compositions define a trend between the low-K_2_O melts at ~ 55 wt% SiO_2_ and high K_2_O melts at > 65 wt% SiO_2_. Therefore, we infer that these interstitial melts represent late stage mixing between silicic melts and less evolved melts. This mixing episode may occur as evolved, hydrous interstitial melts percolate through a crystal mush causing disaggregation of cumulate material into the final erupted magmas. As discussed above, this open-system process may also account for the high K_2_O MI trend. These observations are consistent with the presence of gabbroic clots and amphibole breakdown textures within the volcanic rocks from the Quill (Roobol and Smith 2004). Therefore, the volcanic whole-rock and interstitial melts both suggest that mixing occurred between the plutonic xenolith assemblages and multiple evolved, hydrous melts (both low-K_2_O and high-K_2_O).

### Magmatic plumbing system beneath Statia

The petrology and geochemistry of plutonic xenoliths from Statia provide insights into the structure, and the magmatic processes operating within the sub-volcanic plumbing system. The plutonic xenoliths represent portions of a large zone of volatile-rich, and crystal-rich magmas (or ‘mush’) within the mid-upper crust. In the lower crust, beneath the mush zone, we infer that differentiation of mantle-derived magmas took place. The relatively low Fo contents of Statia olivines suggest melts arriving in the mid-upper crust have undergone prior olivine fractionation. Therefore, the lower crust is likely composed of dense olivine ± clinopyroxene-rich cumulates, that are not sampled as xenoliths on Statia. Melts that segregate from the deep crust then ascend and stall in the mid-upper crustal mush zone. There, a large diversity of trapped melts, ranging from basalts to rhyolites, are generated by crystallisation and melt migration. Variations in phase assemblages and the geochemistry of crystals and melt can be controlled by variations in water content and temperatures (Fig. 12), as well as ƒO_2_, within the crystal mush. The diverse range of melts were likely stored in multiple, variously differentiated melt lenses that were variably mixed prior to final shallow storage and eruption. Variation in melt volatile contents may reflect variations in the volatile budget of input mantle-derived melts, but might equally represent different storage levels, and hence saturation pressures, for input melts with broadly similar volatile contents. The majority of erupted volcanic magmas have a reduced range of compositions in comparison to plutonic MIs, and therefore, the large crystal mush acts as a compositional filter, within which relatively low-variance mineral assemblages, such as cpx + opx + plagioclase + amphibole + oxides, tend to buffer the chemistry of the magmas that emerge from its upper reaches (Blatter et al. 2017). Chemical buffering is most likely when the volume ratio of input melt to mush is low. Final, pre-eruptive storage (or stalling) regions were likely outside of the amphibole stability field, and all amphibole present in the volcanic rocks comes from disaggregated cumulates entrained by ascending melts. Amphibole breakdown reactions, as well as percolation of chemically distinct evolved melts, enriched localised portions of the mush in incompatible elements such as K_2_O, Ba, Sc, and Y. Therefore, non-cumulate orthopyroxene-bearing gabbros, identified by texture, mineral chemistry and whole rock chemistry, represent the plutonic equivalents of the final erupted melts, or frozen aliquots of shallow magma lenses. The mineral phases in non-cumulate gabbros have a more evolved chemistry, similar to the range in mineral chemistry in the volcanic rocks, and strongly zoned plagioclase. Therefore, these samples likely interacted regularly with the final erupted, melt-dominant bodies and may have disaggregated, supplying a crystal cargo to the final erupted magmas (Roobol and Smith, 2004).

## Conclusions

Plutonic xenoliths from Statia provide a unique record of the storage conditions and magmatic processes within the sub-volcanic plumbing system that would not be accessible from studying erupted volcanic rocks alone. This study has revealed the following:

1. There is a large range of xenolith types, mineralogies and textures, with variable crystallisation sequences. These differences can be largely attributed to variations in temperature and pressure within a mid-upper crustal plumbing system. The geochemical characteristics of one group of plutonic xenoliths, the ‘non-cumulate gabbros’, are inferred to represent solidified regions of melt-dominant bodies that were subsequently erupted. In contrast, plutonic xenoliths of cumulate origin are interpreted to represent portions of a large-scale crystal-rich mush.
2. Melt inclusions hosted in Statia plutonic xenoliths indicate that the magmatic plumbing system is volatile rich (≤ 9 wt% H_2_O, 0–1350 ppm CO_2_). Minimum volatile saturation pressures cover a large range 0–426 MPa suggesting crystallisation and/or storage in the upper-middle crust (≤ 15 km depth).
3. Plutonic xenolith MIs preserve an extraordinary range in composition, from basalt to rhyolite providing a record of melt differentiation along liquid lines of descent comparable to experimental studies. Compositions of MIs hosted in different phases are consistent with sample assemblage and crystallisation sequence, with early crystallising olivine hosting the least evolved melts, and later crystallising clinopyroxene and orthopyroxene hosting the most evolved melts. Melts associated with hornblende gabbros describe unusual differentiation trends that may reflect elevated oxidation states within the mush giving rise to early and abundant magnetite crystallisation. This in turn may contribute to the compositional ‘gap’ observed in Statia melt compositions and at other volcanic centres in the Lesser Antilles.
4. The range in melt chemistry of MIs is greater than that of volcanic whole rocks and indicates the Statia plumbing system contains a large diversity of melts which were variably mixed with each other, and with crystal cargos prior to eruption. The plutonic xenoliths provide a means to study the whole range of material present in a magmatic plumbing system, which otherwise would be obscured due to the filtering of melts within a crystal mush.

## Electronic supplementary material

Below is the link to the electronic supplementary material.

Supplementary material 1 (XLSX 318 kb)

Supplementary material 2 (PDF 155928 kb)

Supplementary material 3 (EPS 2277 kb)

## References

[CR1] Arculus RJ, Wills KJA (1980). The petrology of plutonic blocks and inclusions from the Lesser Antilles Island Arc. J Petrol.

[CR2] Balcone-Boissard H, Boudon G, Blundy JD, Martel C, Brooker RA, Deloule E, Solaro C, Matjuschkin V (2018). Deep pre-eruptive storage of silicic magmas feeding Plinian and dome-forming eruptions of central and northern Dominica (Lesser Antilles) inferred from volatile contents of melt inclusions. Contrib Miner Petrol.

[CR3] Barclay J, Rutherford MJ, Carroll MR, Murphy MD, Devine JD, Gardner J, Sparks RSJ (1998). Experimental phase equilibria constraints on pre-eruptive storage conditions of the Soufrière Hills magma. Geophys Res Lett.

[CR4] Beard JS, Ragland PC, Crawford ML (2005). Reactive bulk assimilation: a model for crust-mantle mixing in silicic magmas. Geology.

[CR5] Blatter DL, Sisson TW, Hankins WB (2017). Voluminous arc dacites as amphibole reaction-boundary liquids. Contrib Miner Petrol.

[CR6] Blundy J, Cashman K (2008). Petrologic reconstruction of magmatic system variables and processes. Rev Mineral Geochem.

[CR7] Blundy JD, Holland TJB (1990). Calcic amphibole equilibria and a new amphibole-plagioclase geothermometer. Contrib Miner Petrol.

[CR8] Bouvier A-S, Metrich N, Deloule E (2008). Slab-derived fluids in the magma sources of St. Vincent (Lesser Antilles Arc): volatile and light element imprints. J Petrol.

[CR9] Bouysse P, Westercamp D (1990). Subduction of Atlantic aseismic ridges and Late Cenozoic evolution of the Lesser Antilles island arc. Tectonophysics.

[CR10] Brophy JG (1991). Composition gaps, critical crystallinity, and fractional crystallization in orogenic (calc-alkaline) magmatic systems. Contrib Miner Petrol.

[CR11] Buckley VJE, Sparks RSJ, Wood BJ (2006). Hornblende dehydration reactions during magma ascent at Soufrière Hills Volcano, Montserrat. Contrib Miner Petrol.

[CR12] Camejo-Harry M, Melekhova E, Blundy J, Attridge W, Robertson R, Christopher T (2018). Magma evolution beneath Bequia, Lesser Antilles, deduced from petrology of lavas and plutonic xenoliths. Contrib Mineral Petrol.

[CR13] Camejo-Harry M, Melekhova E, Blundy J, Robertson R (2019). Evolution in magma storage conditions beneath Kick-’em-Jenny and Kick-’em-Jack submarine volcanoes, Lesser Antilles arc. J Volcanol Geoth Res.

[CR14] Cassidy M, Edmonds M, Watt SF, Palmer MR, Gernon TM (2015). Origin of basalts by hybridization in andesite-dominated arcs. J Petrol.

[CR15] Charlier B, Namur O, Toplis MJ, Schiano P, Cluzel N, Higgins MD, Auwera JV (2011). Large-scale silicate liquid immiscibility during differentiation of tholeiitic basalt to granite and the origin of the Daly gap. Geology.

[CR16] Chayes F (1963). Relative abundance of intermediate members of the oceanic basalt-trachyte association. J Geophys Res.

[CR17] Cooper GF, Davidson JP, Blundy JD (2016). Plutonic xenoliths from Martinique, Lesser Antilles: evidence for open system processes and reactive melt flow in island arc crust. Contrib Miner Petrol.

[CR18] Daly RA (1925) The geology of Ascension island. In: Proceedings of the American Academy of Arts and Sciences. JSTOR, pp 3–80

[CR19] Davidson J, Wilson M (2011). Differentiation and source processes at Mt Pelee and the Quill; active volcanoes in the Lesser Antilles Arc. J Petrol.

[CR20] Della-Pasqua FN, Kamenetsky VS, Gasparon M, Crawford AJ, Varne R (1995). Al-spinels in primitive arc volcanics. Mineral Petrol.

[CR21] Dufek J, Bachmann O (2010). Quantum magmatism: magmatic compositional gaps generated by melt-crystal dynamics. Geology.

[CR22] Edmonds M, Kohn SC, Hauri EH, Humphreys MCS, Cassidy M (2016). Extensive, water-rich magma reservoir beneath southern Montserrat. Lithos.

[CR23] Emeleus CH, Cheadle MJ, Hunter RH, Upton BGJ, Wadsworth WJ (1996). The rum layered suite. Developments in petrology.

[CR24] Erdmann M, Koepke J (2016). Silica-rich lavas in the oceanic crust: experimental evidence for fractional crystallization under low water activity. Contrib Miner Petrol.

[CR25] Ghiorso MS, Gualda GA (2015). An H_2_O–CO_2_ mixed fluid saturation model compatible with rhyolite-MELTS. Contrib Miner Petrol.

[CR26] Grove TL, Donnelly-Nolan JM (1986). The evolution of young silicic lavas at Medicine Lake Volcano, California: implications for the origin of compositional gaps in calc-alkaline series lavas. Contrib Miner Petrol.

[CR27] Grove TL, Donnelly-Nolan JM, Housh T (1997). Magmatic processes that generated the rhyolite of Glass Mountain, Medicine Lake volcano, N. California. Contrib Mineral Petrol.

[CR28] Gurenko AA, Trumbull RB, Thomas R, Lindsay JM (2005). A melt inclusion record of volatiles, trace elements and Li–B isotope variations in a single magma system from the Plat Pays Volcanic Complex, Dominica, Lesser Antilles. J Petrol.

[CR29] Harrison TM, Watson EB (1984). The behavior of apatite during crustal anatexis: equilibrium and kinetic considerations. Geochim Cosmochim Acta.

[CR30] Howe TM, Lindsay JM, Shane P (2015). Evolution of young andesitic–dacitic magmatic systems beneath Dominica, Lesser Antilles. J Volcanol Geoth Res.

[CR31] Humphreys MCS, Kearns SL, Blundy JD (2006). SIMS investigation of electron-beam damage to hydrous, rhyolitic glasses: implications for melt inclusion analysis. Am Miner.

[CR32] Humphreys MCS, Edmonds M, Christopher T, Hards V (2009). Chlorine variations in the magma of Soufrière Hills Volcano, Montserrat: insights from Cl in hornblende and melt inclusions. Geochim Cosmochim Acta.

[CR33] Humphreys MCS, Edmonds M, Christopher T, Hards V (2010). Magma hybridisation and diffusive exchange recorded in heterogeneous glasses from Soufrière Hills Volcano. Montserrat. Geophys Res Lett.

[CR34] Jagoutz O, Müntener O, Schmidt MW, Burg J-P (2011). The roles of flux- and decompression melting and their respective fractionation lines for continental crust formation: evidence from the Kohistan arc. Earth Planet Sci Lett.

[CR35] Johnson MC, Rutherford MJ (1989). Experimental calibration of the aluminum-in-hornblende geobarometer with application to Long Valley caldera (California) volcanic rocks. Geology.

[CR36] Johnson ER, Wallace PJ, Cashman KV, Granados HD, Kent AJ (2008). Magmatic volatile contents and degassing-induced crystallization at Volcán Jorullo, Mexico: implications for melt evolution and the plumbing systems of monogenetic volcanoes. Earth Planet Sci Lett.

[CR37] Kawamoto T (1996). Experimental constraints on differentiation and H_2_O abundance of calc-alkaline magmas. Earth Planet Sci Lett.

[CR38] Kiddle E. J., Edwards B. R., Loughlin S. C., Petterson M., Sparks R. S. J., Voight B. (2010). Crustal structure beneath Montserrat, Lesser Antilles, constrained by xenoliths, seismic velocity structure and petrology. Geophysical Research Letters.

[CR39] Leake BE, Woolley AR, Birch WD, Burke EA, Ferraris G, Grice JD, Hawthorne FC, Kisch HJ, Krivovichev VG, Schumacher JC (2003). Nomenclature of amphiboles: additions and revisions to the International Mineralogical Association’s 1997 recommendations. Can Mineral.

[CR40] Lee C-TA, Morton DM (2015). High silica granites: terminal porosity and crystal settling in shallow magma chambers. Earth Planet Sci Lett.

[CR41] Liu Y, Anderson AT, Wilson CJ, Davis AM, Steele IM (2006). Mixing and differentiation in the Oruanui rhyolitic magma, Taupo, New Zealand: evidence from volatiles and trace elements in melt inclusions. Contrib Mineral Petrol.

[CR42] Macdonald R, Hawkesworth CJ, Heath E (2000). The Lesser Antilles volcanic chain: a study in arc magmatism. Earth Sci Rev.

[CR43] Mann CP, Wallace PJ, Stix J (2013). Phenocryst-hosted melt inclusions record stalling of magma during ascent in the conduit and upper magma reservoir prior to vulcanian explosions, Soufrière Hills volcano, Montserrat, West Indies. Bull Volcanol.

[CR44] Marsh BD (1981). On the crystallinity, probability of occurrence, and rheology of lava and magma. Contrib Mineral Petrol.

[CR45] Martel C, Pichavant M, Bourdier J-L, Traineau H, Holtz F, Scaillet B (1998). Magma storage conditions and control of eruption regime in silicic volcanoes: experimental evidence from Mt. Pelée. Earth Planet Sci Lett.

[CR46] Martel C, Pichavant M, Holtz F, Scaillet B, Bourdier J-L, Traineau H (1999). Effects of f O_2_ and H_2_O on andesite phase relations between 2 and 4 kbar. J Geophys Res: Solid Earth.

[CR47] Melekhova E, Annen C, Blundy J (2013). Compositional gaps in igneous rock suites controlled by magma system heat and water content. Nat Geosci.

[CR48] Melekhova E, Blundy J, Robertson R, Humphreys MC (2015). Experimental evidence for polybaric differentiation of primitive arc basalt beneath St. Vincent, Lesser Antilles. J Petrol.

[CR49] Melekhova E, Blundy J, Martin R, Arculus R, Pichavant M (2017). Petrological and experimental evidence for differentiation of water-rich magmas beneath St. Kitts. Lesser Antilles. Contrib Mineral Petrol.

[CR50] Melekhova E, Schlaphorst D, Blundy J, Kendall J-M, Connolly C, McCarthy A, Arculus R (2019). Crustal structure variation along the Lesser Antilles arc inferred from seismology and petrology. Earth Planet Sci Lett.

[CR51] Moore LR, Gazel E, Tuohy R, Lloyd AS, Esposito R, Steele-MacInnis M, Hauri EH, Wallace PJ, Plank T, Bodnar RJ (2015). Bubbles matter: an assessment of the contribution of vapor bubbles to melt inclusion volatile budgets. Am Mineral.

[CR52] Müntener O, Ulmer P (2018). Arc crust formation and differentiation constrained by experimental petrology. Am J Sci.

[CR53] Nandedkar RH, Ulmer P, Müntener O (2014). Fractional crystallization of primitive, hydrous arc magmas: an experimental study at 0.7 GPa. Contrib Mineral Petrol.

[CR54] Newman S, Lowenstern JB (2002). VolatileCalc: a silicate melt–H2O–CO2 solution model written in Visual Basic for excel. Comput Geosci.

[CR55] O’Driscoll B, Donaldson CH, Troll VR, Jerram DA, Emeleus CH (2006). An origin for harrisitic and granular olivine in the rum layered suite, NW Scotland: a crystal size distribution study. J Petrol.

[CR56] Palme H, O’Neill HSC (2003). Cosmochemical estimates of mantle composition. Treatise Geochem.

[CR57] Pichavant M, Macdonald R (2007). Crystallization of primitive basaltic magmas at crustal pressures and genesis of the calc-alkaline igneous suite: experimental evidence from St Vincent, Lesser Antilles arc. Contrib Mineral Petrol.

[CR58] Pichavant M, Martel C, Bourdier J-L, Scaillet B (2002) Physical conditions, structure, and dynamics of a zoned magma chamber: Mount Pelée (Martinique, Lesser Antilles Arc). J Geophys Res: Solid Earth 107, B5, 2093

[CR59] Pichavant M, Mysen BO, Macdonald R (2002). Source and H_2_O content of high-MgO magmas in island arc settings: an experimental study of a primitive calc-alkaline basalt from St. Vincent, Lesser Antilles arc. Geochimica et Cosmochimica Acta.

[CR60] Pichavant M, Di Carlo I, Le Gac Y, Rotolo SG, Scaillet B (2009). Experimental constraints on the deep magma feeding system at Stromboli Volcano, Italy. J Petrology.

[CR61] Pichavant M, Carlo ID, Rotolo SG, Scaillet B, Burgisser A, Gall NL, Martel C (2013). Generation of CO_2_-rich melts during basalt magma ascent and degassing. Contrib Mineral Petrol.

[CR62] Putirka KD (2008). Thermometers and barometers for volcanic systems. Rev Mineral Geochem.

[CR63] Reubi O, Blundy J (2008). Assimilation of plutonic roots, formation of High-K ‘Exotic’ Melt Inclusions and Genesis of Andesitic Magmas at Volcán De Colima, Mexico. J Petrol.

[CR64] Reubi O, Blundy J (2009). A dearth of intermediate melts at subduction zone volcanoes and the petrogenesis of arc andesites. Nature.

[CR65] Roberge J, Delgado-Granados H, Wallace PJ (2009). Mafic magma recharge supplies high CO_2_ and SO_2_ gas fluxes from Popocatépetl volcano, Mexico. Geology.

[CR66] Roedder E (1984). Fluid inclusions. Rev Mineral.

[CR67] Roobol MJ, Smith AL (2004) Volcanology of Saba and St. Eustatius, Northern Lesser Antilles. Koninklijke nederlandse Akademie van wetenschappen

[CR68] Rutherford MJ, Hill PM (1993). Magma ascent rates from amphibole breakdown: an experimental study applied to the 1980–1986 Mount St. Helens eruptions. J Geophys Res: Solid Earth.

[CR69] Schiano P, Clocchiatti R, Boivin P, Medard E (2004). The nature of melt inclusions inside minerals in an ultramafic cumulate from Adak volcanic center, Aleutian arc: implications for the origin of high-Al basalts. Chem Geol.

[CR70] Stamper CC, Blundy JD, Arculus RJ, Melekhova E (2014). Petrology of plutonic xenoliths and volcanic rocks from Grenada, Lesser Antilles. J Petrol.

[CR71] Streckeisen A (1976). To each plutonic rock its proper name. Earth Sci Rev.

[CR72] Sugawara Toru (2001). Ferric iron partitioning between plagioclase and silicate liquid: thermodynamics and petrological applications. Contrib Mineral Petrol.

[CR73] Tollan PME, Bindeman I, Blundy JD (2012). Cumulate xenoliths from St. Vincent, Lesser Antilles Island Arc: a window into upper crustal differentiation of mantle-derived basalts. Contrib Mineral Petrol.

[CR74] Wadsworth WJ (1961). The layered ultrabasic rocks of south-west Rhum, Inner Hebrides. Philos Trans R Soc Lond B.

[CR75] Wager LR, Brown GM, Wadsworth WJ (1960). Types of igneous cumulates. J Petrol.

[CR76] Wallace PJ (2005). Volatiles in subduction zone magmas: concentrations and fluxes based on melt inclusion and volcanic gas data. J Volcanol Geotherm Res.

[CR77] Wallace PJ, Anderson AT, Davis AM (1999). Gradients in H_2_O, CO_2_, and exsolved gas in a large-volume silicic magma system: interpreting the record preserved in melt inclusions from the Bishop Tuff. J Geophys Res: Solid Earth.

[CR78] Watson EB, Harrison TM (1983). Zircon saturation revisited: temperature and composition effects in a variety of crustal magma types. Earth Planet Sci Lett.

[CR79] Webster JD, Rebbert CR (2001). The geochemical signature of fluid-saturated magma determined from silicate melt inclusions in Ascension Island granite xenoliths. Geochimica et Cosmochimica Acta.

[CR80] Westermann JH, Kiel H (1961) The Geology of Saba and St. Eustatius: with notes on the geology of St. Kitts, Nevis and Montserrat (Lesser Antilles). Foundation for scientific research in Surinam and the Netherlands Antilles, 24

[CR81] Yanagida Y, Nakamura M, Yasuda A, Kuritani T, Nakagawa M, Yoshida T (2018). Differentiation of a hydrous arc magma recorded in melt inclusions in deep crustal cumulate xenoliths from Ichinomegata Maar, NE Japan. Geochem Geophys Geosyst.

[CR82] Ziberna L, Green EC, Blundy JD (2017). Multiple-reaction geobarometry for olivine-bearing igneous rocks. Am Mineral.

