# Supplementary figures and images for: Evidence from plutonic xenoliths for magma differentiation, mixing and storage in a volatile-rich crystal mush beneath St. Eustatius, Lesser Antilles
Source: Contrib Mineral Petrol. 2019 May 6;174(5):39. doi: 10.1007/s00410-019-1576-4 (PMC6530818; doi:10.1007/s00410-019-1576-4)

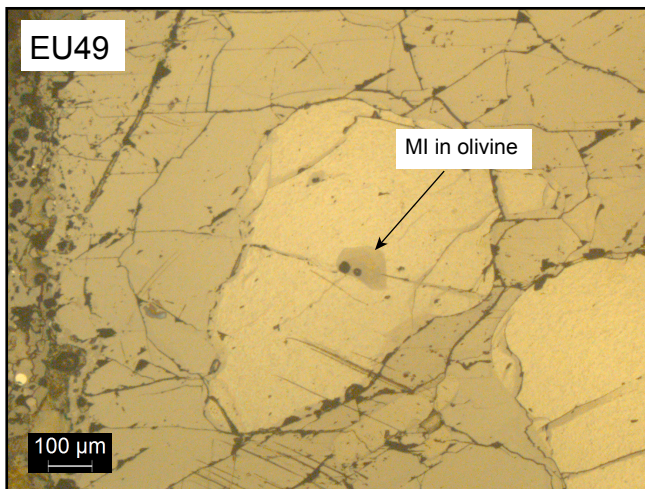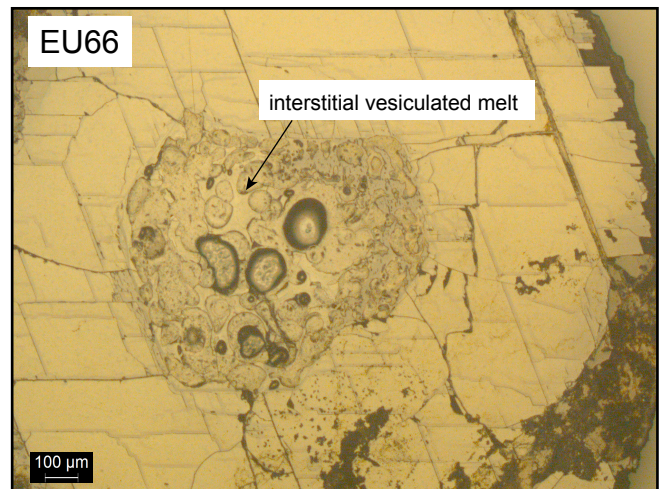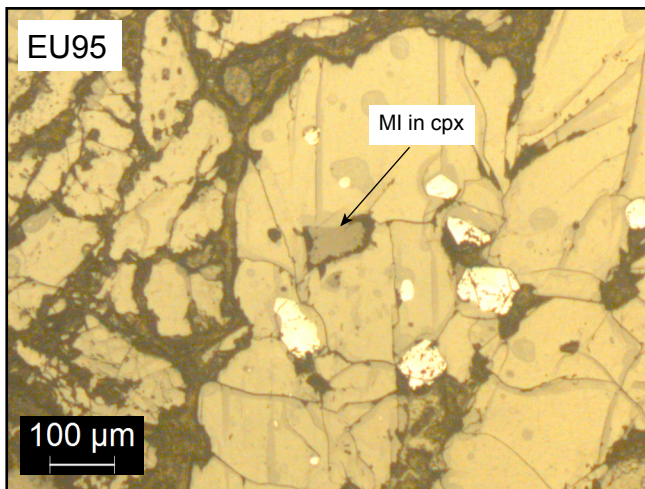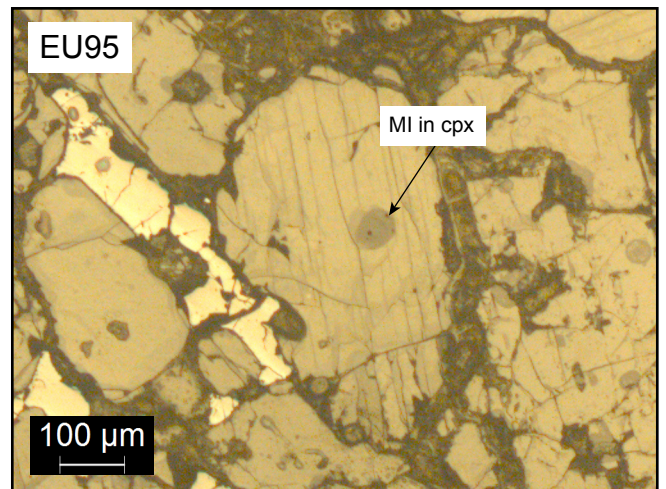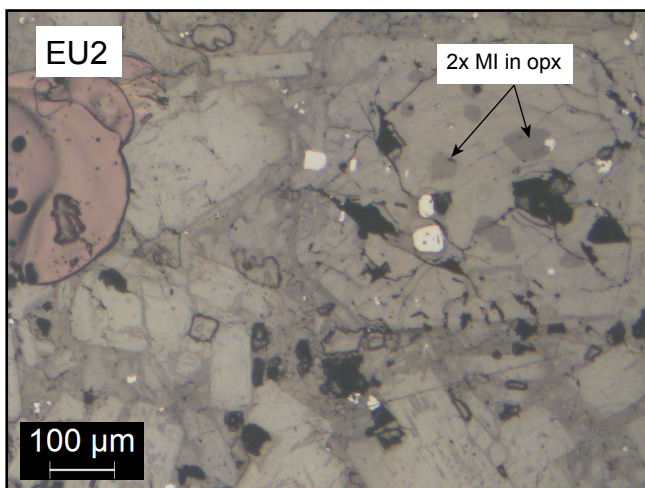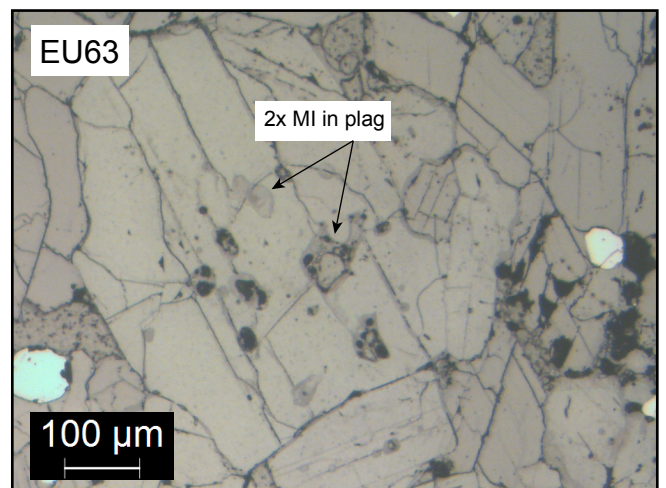

Reflected light images of cumulate hosted melt inclusions and interstitial melt.

Supplement: Supplementary file 2 — Supplementary material 2 (PDF 155928 kb) [file 410_2019_1576_MOESM2_ESM.pdf]
